# Computational electrostatic engineering of nanobodies for enhanced SARS−CoV−2 receptor binding domain recognition

**DOI:** 10.3389/fmolb.2025.1512788

**Published:** 2025-03-10

**Authors:** Zafar Iqbal, Muhammad Asim, Umair Ahmad Khan, Neelam Sultan, Irfan Ali

**Affiliations:** ^1^ Central Laboratories, King Faisal University, Al Hofuf, Saudi Arabia; ^2^ Centre of Agricultural Biochemistry and Biotechnology (CABB), University of Agriculture, Faisalabad, Pakistan; ^3^ Medical and Allied Department, Faisalabad Medical University, Faisalabad, Pakistan; ^4^ Department of Biochemistry, Government College University Faisalabad, Faisalabad, Pakistan

**Keywords:** electrostatic potential, nanobodies, ACE-2 receptor, SARS-CoV-2, spike protein, receptor binding domain, molecular docking simulation

## Abstract

This study presents a novel computational approach for engineering nanobodies (Nbs) for improved interaction with receptor-binding domain (RBD) of the SARS-CoV-2 spike protein. Using Protein Structure Reliability reports, RBD (7VYR_R) was selected and refined for subsequent Nb-RBD interactions. By leveraging electrostatic complementarity (EC) analysis, we engineered and characterized five Electrostatically Complementary Nbs (ECSb1-ECSb5) based on the CeVICA library’s SR6c3 Nb. Through targeted modifications in the complementarity-determining regions (CDR) and framework regions (FR), we optimized electrostatic interactions to improve binding affinity and specificity. The engineered Nbs (ECSb3, ECSb4, and ECSb5) demonstrated high binding specificity for AS3, CA1, and CA2 epitopes. Interestingly, ECSb1 and ECSb2 selectively engaged with AS3 and CA1 instead of AS1 and AS2, respectively, due to a preference for residues that conferred superior binding complementarities. Furthermore, ECSbs significantly outperformed SR6c3 Nb in MM/GBSA results, notably, ECSb4 and ECSb3 exhibited superior binding free energies of −182.58 kcal.mol^-1^ and −119.07 kcal.mol^-1^, respectively, compared to SR6c3 (−105.50 kcal.mol^-1^). ECSbs exhibited significantly higher thermostability (100.4–148.3 kcal·mol⁻^1^) compared to SR6c3 (62.6 kcal·mol⁻^1^). Similarly, enhanced electrostatic complementarity was also observed for ECSb4-RBD and ECSb3-RBD (0.305 and 0.390, respectively) relative to SR6c3-RBD (0.233). Surface analyses confirmed optimized electrostatic patches and reduced aggregation propensity in the engineered Nb. This integrated EC and structural engineering approach successfully developed engineered Nbs with enhanced binding specificity, increased thermostability, and reduced aggregation, laying the groundwork for novel therapeutic applications targeting the SARS-CoV-2 spike protein.

## 1 Introduction

The COVID−19 pandemic, caused by SARS-CoV-2, created a global health crisis of unprecedented magnitude, overwhelming healthcare systems (Ranney et al., 2020) and leading to substantial loss of life (Wu and McGoogan, 2020), financial strain (Nicola et al., 2020), unemployment (Coibion et al., 2021), and widespread supply chain disruptions (Golan et al., 2020). Additionally, the pandemic highlighted social and economic disparities, with marginalized communities often bearing a disproportionate burden of the virus’s impact (Egede and Walker, 2020; Tai et al., 2021). While posing immense challenges, the pandemic also spurred innovation in vaccine development (Krammer, 2020) and restructuring of work environments (Kniffin et al., 2021), reshaping the future of global health and society. Notwithstanding this progress, the virus’s swiftly evolving nature necessitated a prompt and pressing need for research on efficacious therapies to curb emerging/evolving strains and mitigate the pandemic’s ongoing impact (Graham, 2020). Developing such therapies entails an understanding of the molecular mechanisms underlying SARS−CoV−2 infection, particularly the role of the spike (S) protein in facilitating viral entry into host cells.

The S protein comprises S1 and S2 subunits, with S2 containing highly conserved regions (Premkumar et al., 2020). A successful SARS-CoV-2 infection hinges on the specific binding of the viral spike (S) protein to cellular entry receptors, primarily the angiotensin−converting enzyme 2 (ACE2) (Lan et al., 2020; Shang et al., 2020; Walls et al., 2020; Wang et al., 2020; Zhou et al., 2020; V’kovski et al., 2021). This receptor engagement has been a key factor in the virus’s adaptation from an animal reservoir and its ongoing evolution (Letko et al., 2020). The immune system combats SARS−CoV−2 by generating a diverse array of antibodies that target various viral epitopes, thereby inhibiting cellular entry, and by activating T cells that eliminate infected host cells. Monoclonal antibodies (mAbs) targeting conserved regions of the S1 or S2 subunits, such as hmAb 1249A8, S2P6, and CC40.8, have demonstrated broad efficacy against SARS-CoV-2 variants (Tian et al., 2020; Cameroni et al., 2021; Chen P. et al., 2021; Starr et al., 2021; Park et al., 2022). These mAbs can directly interfere with viral pathogenesis by neutralizing virions, opsonizing infected cells, or inducing cell death. While mAbs offer broad efficacy, their widespread use is hindered by high production costs (Flaxman et al., 2022; Popping et al., 2023) and the risk of repeated viral evasion (Liu et al., 2021; Wang Q. et al., 2022). Therefore, alternative strategies are needed to address these challenges and develop more affordable and sustainable countermeasures against SARS−CoV−2 infection.

Nanobodies (Nbs), as single−domain antibodies (Hamers-Casterman et al., 1993), offer several distinct advantages over mAbs, including reduced size, enhanced stability, lower immunogenicity, and streamlined manufacturing process (Muyldermans, 2013; Steeland et al., 2016). Their therapeutic potential has garnered increasing attention, particularly following the FDA’s approval of the first Nb therapeutic in 2019 (Morrison, 2019). The superior stability of a Nb arises from their compact, single−domain structure and their highly polar surface. Nevertheless, achieving optimal Nb performance requires careful optimization of their binding affinity and stability (Kunz et al., 2018; Esparza et al., 2020) which are influenced by a sophisticated interplay of factors, including hydrogen bonding, hydrophobic interactions, van der Waals forces, and electrostatic interactions (Chakrabarti and Janin, 2002; Dellisanti et al., 2007; Boehr et al., 2009; Kastritis and Bonvin, 2013). This intricate balance of forces orchestrates robust and highly selective protein complex formation, underscoring their profound significance given that approximately 80% of proteins function within such assemblies (Gavin et al., 2002; de Las Rivas and Fontanillo, 2010).

Collectively, these forces define binding properties by establishing multiple layers of complementarity—surface (Sc), hydrophobic, and electrostatic (EC)—between the interacting entities (Korn and Burnett, 1991; Braden and Poljak, 1995; Meyer et al., 1996; McCoy et al., 1997; Sinha and Smith-Gill, 2002; Basu and Biswas, 2018). Sc arises from the precise geometric fit of molecules facilitated by van der Waals forces and hydrogen bonding (Chothia and Janin, 1975), while hydrophobic complementarity is driven by the association of nonpolar side chains through hydrophobic interactions (Richards, 1977). Conversely, the alignment of opposite surface charges establishes EC, which plays a pivotal role in protein−protein interactions (PPIs) (McCoy et al., 1997; Sheinerman et al., 2000). EC facilitates the initial encounter between binding partners through long−range electrostatic attractions, effectively steering them toward the correct orientation for binding (Vijayakumar et al., 1998; Sheinerman et al., 2000). This directed guidance reduces the entropic barrier associated with random collisions, thereby increasing the likelihood of productive interactions (Schreiber and Fersht, 1995; Selzer et al., 2000). Furthermore, electrostatic interactions enhance binding strength by forming salt bridges and hydrogen bonds at the interface, augmenting both affinity and specificity (Baker and Hubbard, 1984; Jones and Thornton, 1996; Xu et al., 1997). By stabilizing the transition state and lowering the activation energy required for complex formation, EC significantly contributes to the robustness and efficacy of PPIs (Vijayakumar et al., 1998; Sheinerman et al., 2000). Recognizing its critical role, it becomes essential to explore innovative strategies that leverage EC to enhance the binding properties of therapeutic molecules such as Nbs.

However, despite the significant EC role, previous computational approaches for affinity maturation of Nbs targeting the SARS−CoV−2 RBD have primarily relied on two strategies: (1) grafting complementarity−determining regions (CDRs) from neutralizing Abs onto stable scaffold frameworks (Cao et al., 2020; Wu et al., 2020a; Yang et al., 2021; 2023; Gopal et al., 2022; Rangel et al., 2022; Ferraz et al., 2024), and (2) structure−based site−mutagenesis of CDR amino acids within Nb-RBD docked complexes (Laroche et al., 2022; Longsompurana et al., 2023; Zhu et al., 2023; Hannula et al., 2024; Singh et al., 2024). To the best of our knowledge, the simultaneous engineering of CDRs and FRs has not been previously explored, as prior research has typically focused on modifying either CDRs or FRs independently to enhance binding affinity through electrostatic attractions (Sinha et al., 2002; Fukunaga and Tsumoto, 2013; Kiyoshi et al., 2014; Du Q. et al., 2019; Yoshida et al., 2019; Nguyen et al., 2021). This novel approach allows for a more comprehensive optimization of Nb interactions with the SARS−CoV−2 RBD, potentially can lead to the development of more effective therapeutic agents. Leveraging computational techniques, we have predicted the Nbs with enhanced paratope−epitope EC employing this methodology. Unlike earlier EC enhancing studies that primarily targeted binding affinity, our approach systematically evaluates the impact of this strategy on key parameters—including binding interfaces, binding free energy, thermostability, aggregation propensity, and post−translational modification reactivity—with the objective of predicting Nbs with superior binding affinity and stability. To substantiate our approach, we conducted a comparative analysis with the well−characterized Nb SR6c3 from the CeVICA library, which is renowned for its robust neutralizing ability and high thermal stability. Our results on Nb engineering with predicted improvement provide a computational framework for developing effective Nbs against SARS−CoV−2. This approach can offer a promising avenue for engineering of novel and effective therapeutic options by optimizing Sc and hydrophobic complementarity.

## 2 Materials and methods

### 2.1 Sequence retrieval and RBD structure analysis

A dataset of ten RBD structures, including 7EAM_A (Li et al., 2021), 7EAM_B (Li et al., 2021), 7B0B_F (Korenkov et al., 2023), 7BZ5_A (Wu et al., 2020b), 6M0J_E (Lan et al., 2020), 7VYR_C (Jeong et al., 2022), 7VYR_R (Jeong et al., 2022), 7BNV_A (Bullen et al., 2021), 8EL2_A (Hermet et al., 2023), and 8EL2_B (Hermet et al., 2023), was retrieved from the Protein Data Bank (PDB). These structures pre−processed employing the Protein Preparation Wizard (PPW) (Madhavi Sastry et al., 2013; Schrödinger, 2023e) with default settings in BioLuminate (Beard et al., 2013; Salam et al., 2014; Zhu et al., 2014; Schrödinger, 2023a) to ensure optimal quality. Subsequently, Protein Structure Reliability (PSR) analysis was performed to assess most suitable structure for the subsequent energy minimization and loop refinement using Prime module (Jacobson et al., 2002; Jacobson et al., 004; Schrödinger, 2023d) employing the VSGB solvation model (Li et al., 2011) and the OPLS3e force field (Roos et al., 2019). This was achieved by specifically addressing the amino acids that displayed high−temperature factors, peptide planarity problems, or steric clashes while maintaining the original sequence as provided. Finally, the structures were further validated through PSR, Structure Quality Reports (SQR), and Ramachandran plot analysis (Ramachandron et al., 1963).

### 2.2 Epitope selection and their biophysical analysis

**FIGURE 1:**
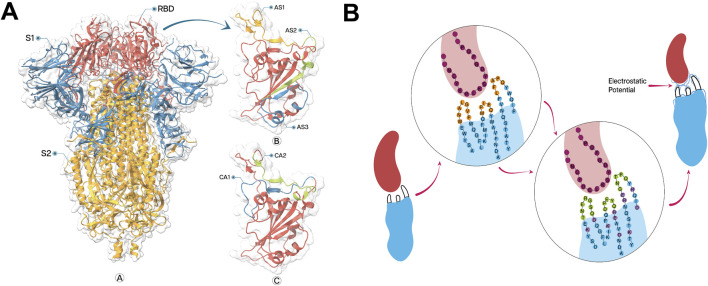
**(A)** Structure of SARS−CoV−2 spike protein (ribbon representation) and neutralizing epitopes on receptor binding domain (RBD), visualized by ChimeraX 1.8. Spike protein structure labelled with sub−domains S1 (Blue) and S2 (Golden) with particular indication of RBD (Red) **(A)**. Enlarged view of the RBD showing the locations of three neutralizing epitopes: AS1, AS2, and AS3 **(B)**. Additional view of RBD highlighting two more recently identified epitopes: CA1 and CA2 (C). **(B)** Integrated approach of CDR designing and FR engineering to develop a greater electrostatic complementarity. Initial nanobody (blue) and antigen (red) interaction (left) with less EC against epitope with fewer counterpart charged amino acids in CDRs (orange) and FRs (blue). The circular inset shows the amino acid sequence at the binding interface, with the antigen sequence in purple and the nanobody sequence in blue and orange. Modified nanobody with optimized amino acid sequence (Centre) with complete randomization of amino acids in CDRs (green) with engineered FRs (lavender) generating a greater EC against epitope with greater electrostatic potential (blue lines on CDRs).

We focused on three previously reported neutralizing epitopes, AS1 (Hansen et al., 2020; Pinto et al., 2020), AS2 (Hansen et al., 2020; Robbiani et al., 2020), and AS3 (Yuan et al., 2020), which are established binding sites for single−chain variable fragment (scFv) antibodies (Figure 1A). Additionally, we expanded our repertoire by incorporating two more recently reported immunoglobulin G (IgG)−targeted epitopes, CA1 and CA2 (Contreras et al., 2023). This comprehensive approach leverages both well−studied and newly discovered epitopes to maximize the potential for effective neutralization. To comprehensively characterize the selected epitopes, a detailed analysis of their titration curves and ionizable residues was conducted in BioLuminate. The protonation states of these ionizable residues were determined by employing PROPKA (Li et al., 2005; Bas et al., 2008; Olsson et al., 2011) with default parameters, ensuring accurate representation of the electrostatic properties within the molecular environment.

### 2.3 Complementarity−determining regions (CDR) designing

SR6c3, a CeVICA Nb with IC₅₀ of 62.7 nM and melting temperature of 72°C, was selected as the reference Nb for the current study due to its robust neutralizing capability and highest thermal stability within the Nb library (Chen X. et al., 2021). Although the monomeric form of SR6v15 exhibits a superior inhibitory potency with an IC₅₀ of 2.18 nM, SR6c3 was preferred for its exceptional thermal resilience coupled with substantial neutralizing efficacy. SR6v15 demonstrated lower refolding capacity, with a heated/non−heated ratio of 0.72, in contrast to SR6c3, which achieved a ratio greater than one indicating an enhanced binding affinity following complete thermal denaturation at 98°C and refolding. Therefore, SR6c3 was chosen based on its superior performance.

Using standard frame sequences of SR6c3 as scaffolds, we designed CDRs to enhance EC between the paratope and the epitope, by prioritizing residues with opposing charges and significant sidechain pKa. For neutral epitope residues, polar amino acids with high free energies of association were chosen (Du H. et al., 2019). CDR1 and CDR2 lengths were constrained to 5-7 amino acids to prevent the potential structural instability, while CDR3 length was allowed to vary randomly. The incorporation of cysteine residues, a defining characteristic of the CeVICA library, was also considered during CDR design (Figure 1B). ColabFold v1.5.2 (Mirdita et al., 2022) was used, with default settings, to predict the structures of five Nbs involving CDR design, alongside the reference Nb SR6c3.

### 2.4 Framework regions (FR) engineering

The initial Nb structures were subjected to pre-processing using the PPW program with the default settings (Madhavi Sastry et al., 2013; Schrödinger, 2023e) in BioLuminate (Beard et al., 2013; Salam et al., 2014; Zhu et al., 2014; Schrödinger, 2023a). Unlike traditional sequence-based CDR designing approaches, our strategy for engineering the FRs of SR6c3 was essentially structure−based. Pre−processed Nbs were carefully examined using BioLuminate to identify specific residues that are suitable for mutation, as proposed by Fukunaga et al., (Fukunaga and Tsumoto, 2013). A selection range of five residues was defined in the vicinity of the CDRs from FR1, FR2, and FR4, while for longer FR3, this range was extended to eight. It is noteworthy that FR2 hallmark residues (F37, E44, R45, and V48) were deliberately left un-mutated due to their crucial role in the independence of the light chain (Muyldermans et al., 1994; Padlan, 1994; Vu et al., 1997; Muyldermans, 2013).

We refined our residue selection process based on Fukunaga et al., (Fukunaga and Tsumoto, 2013), targeting residues adjacent to CDRs, exposed to the molecular surface, and with sidechains at least 20% accessible to the solvent to ensure the relevance of our mutation targets. Following these specified criteria, we analyzed the electrostatic topography of the RBD and epitopes, then formulated a mutation strategy to introduce charged amino acids into each Nb structure. The initial phase of the strategy involved mutating the neighboring residues around the CDRs to amplify EC between the CDRs region and the epitope, while mutating outlying residues to improve the overall EC between Nb and RBD. Furthermore, we substituted specific hydrophobic residues with polar ones to reduce aggregation and enhance solubility (Figure 1B).

For the computational evaluation of Nbs’ humanization profiles, AbNatiV (Ramon et al., 2024), a deep learning tool was used with default settings. The amino acid sequences of the newly designed Nbs and SR6c3 were submitted to AbNatiV, utilizing the VHH domain mode. However, alignment with CeVICA revealed the adoption of the same VHH hallmark residues (F37, E44, R45, and V48) *in lieu* of human residues, reflecting their advantageous role in post−minus pre−affinity maturation (Chen X. et al., 2021). ColabFold v1.5.2 (Mirdita et al., 2022) was then employed with default settings to predict the structural conformations of the five Electrostatically Complementary Nbs (ECSbs) and SR6c3 based on their consensus sequences.

### 2.5 Structural refinement and analyses

Initial structural refinement of SR6c3 and ECSbs was performed using PPW (Madhavi Sastry et al., 2013; Schrödinger, 2023e) with default settings in BioLuminate (Beard et al., 2013; Salam et al., 2014; Zhu et al., 2014; Schrödinger, 2023a), addressing potential issues like steric clashes, peptide planarity, temperature factors, and dihedral angles. Each ECSb then underwent a meticulous PSR assessment, resulting in the selection of SR6c3, ECSb2, ECSb3, and ECSb5 for subsequent surface analyses. ECSb1 and ECSb4 required additional energy minimizations and loop refinement at specific amino acids, which were conducted using Prime employing the VSGB solvation model (Li et al., 2011) and the OPLS3e force field (Roos et al., 2019), followed by PSR reassessment and structure finalization (Jacobson et al., 2002; 2004; Schrödinger, 2023d). All six structures were further evaluated using SQRs and Ramachandran plots (Ramachandran et al., 1963). Post evaluation, an exhaustive analysis of electrostatically charged patches, aggregation scores using AggScore (Sankar et al., 2018), and residues reactivity profiles of each structure was conducted. These comprehensive analyses, performed using BioLuminate’s protein surface analysis tool, were subsequently compared with analogous analyses of SR6c3. Following the surface analyses, the FoldX Suite 4.0 (Schymkowitz et al., 2005), integrated as a YASARA View (Krieger and Vriend, 2014), was employed for the computation of the thermal stability of Nbs. To further investigate the grounds underneath the enhanced stability of ECSbs compared to SR6c3, we utilized ProDy’s InSty module (Bakan et al., 2011; Bakan et al., 2014; Zhang et al., 2021). Using default parameters (distance, angle, and cutoff distance), we analyzed seven types of intramolecular interactions, including hydrogen bonds (HBs), salt bridges (SBs), repulsive ionic interactions (RIB), hydrophobic interactions (HPh), disulfide bonds (DiBs), π−stacking (Pistacking), and π−cation (PiCation) interactions. Additionally, key residues with significant contributions to these interactions were identified, particularly those playing prominent roles in the interaction network of each Nb.

### 2.6 Protein-protein docking

To identify potential binding sites on the pre-processed RBD for Nbs (ECSbs and SR6c3), we initially employed an FFT-based global docking approach using PIPER within BioLuminate (Kozakov et al., 2006; Chuang et al., 2008; Fernández-Recio and Sternberg, 2010; Desta et al., 2020; Schrödinger, 2023c). This procedure generated 30 unique conformations for each RBD-Nb complex. Due to the inherent rigidity of PIPER’s docking methodology, each docked pose was pre-processed before calculating the binding free energy using the Molecular Mechanics Generalized Born Surface Area (MM/GBSA) method (Genheden and Ryde, 2015). To identify the most optimal pose between the Nbs and RBD, binding free energy calculations were performed, which subsequently provided the foundation for in-depth interaction analysis and subsequent MD simulations.

To visually validate the EC of the binding interfaces in Nb-RBD complexes, we utilized the APBS (Adaptive Poisson−Boltzmann Solver) (Jurrus et al., 2018) plugin within PyMOL version 3.1.1 (Schrodinger, 2024) to generate and visualize electrostatic potential maps. Molecular structures were prepared using PDB2PQR (Dolinsky et al., 2004; 2007) with default parameters. Electrostatic potentials were calculated over a range of ±2.0 kcal.mol^−1^·*e*, employing a solvent-excluded surface and a grid spacing of 0.50 Å.

The rigidity of FFT-based approach may limit conformational sampling; therefore, we subsequently employed the Rosetta flexible docking module (Gray et al., 2003; Wang et al., 2005; Chaudhury and Gray, 2008; Marze et al., 2018). This flexible protocol refines both backbone and side-chain conformations. Accordingly, all structures were meticulously prepared to ensure optimal backbone and side-chain integrity. Preparation involved reassigning chain IDs: Chain A was designated for RBD, previously labeled as Chain R, while Chain B was assigned to Nb, formerly designated as Chain A. The residues were then renumbered so that the RBD encompassed residues 1–186 and the Nb commenced from residue 187 onward. Subsequently cleaning each PDB file to preserve only standard atomic records, then assessing initial energetic properties with the Rosetta Energy Function 2015 (REF 2015) (Alford et al., 2017). To relieve steric clashes without disrupting the overall fold, a constrained FastRelax protocol was applied (Nivón et al., 2013). Afterward, interfacial residues were identified using a 6.0 Å distance criterion, enabling selective side−chain repacking confined to the interface via the PackRotamersMover with REF 2015. The prepacked structures were subsequently used for further steps.

Docking was conducted using the Rosetta protein docking module to generate 1000 decoys for each complex, wherein the RBD and Nbs were imported and subjected to FastRelax to achieve local geometric optimization. The optimized structures were subsequently assembled into a single pose by introducing a jump, preserving the native configurations derived from the PIPER-docked conformations. Interfacial residues were identified, and side-chain repacking was selectively confined to these regions to maintain the native-like conformation of the remaining protein structure, thereby minimizing structural perturbations outside the binding interface. A final relaxation phase was applied to further refine both side-chain and backbone orientations, resulting in a set of convergent RBD−Nb complexes. For each docking trajectory, the RMSD was calculated based on the heavy atoms of the interface residues, using the PIPER-docked poses as reference structures. This analysis was conducted utilizing the Rosetta InterfaceAnalyzerMover (Benjamin Stranges and Kuhlman, 2013). To characterize the docking funnel, the interface score (I_score) was plotted against the interface RMSD (I_rmsd), facilitating the identification of energetically favorable conformations.

The top-ranked decoys, exhibiting optimal binding energetics and structural stability, were selected for subsequent post−docking analyses. Post-docking analyses included the calculation of binding free energy (ddG), calculations of solvent-accessible surface area (SASA), and evaluations of Sc and EC. The ddG of each bound complex was recalculated using REF 2015 (Alford et al., 2017), and the change in SASA upon complex formation (dSASA) was determined. Shape and electrostatic complementarity (EC) were assessed using the Shape Complementarity Calculator and Electrostatic Complementarity Calculator, providing insights into the geometric fit and electrostatic compatibility at the interface. Additionally, interface residues were identified using the Interface Analyzer Mover in PyRosetta 4.0 (Chaudhury et al., 2010), which is configured to analyze designated chains by computing packing statistics and dSASA. Residues are classified as interface residues based on distance−based contact criteria, with both the total count and the specific set of residues defining the interface extracted from the analysis. Subsequently, the interaction types between these partnered residues are determined by generating a Pose object from the PDB structure and applying the Interface Analyzer Mover (Benjamin Stranges and Kuhlman, 2013) to evaluate intermolecular interactions. In this step, hydrogen bonding and van der Waals contributions were quantified using the HB and LJ Att/LJ Rep energy terms, respectively; salt bridges are identified from the Elec, while π-π, π-cation, and π-anion interactions are inferred from the side−chain geometry and relative orientation of residues. Finally, an interaction network was constructed by parsing dataset of residue-residue interaction data with Pandas and building a graph using NetworkX, where residues serve as nodes and their interactions as edges.

### 2.7 Molecular dynamics simulations

MD simulations were conducted to assess the stability of I_score based top ranked decoys of each Nbs-RBD complex using Desmond (Banks et al., 2005) software within the Schrodinger suite. Nbs-RBD complexes were prepared, optimized, and minimized using the PPW (Madhavi Sastry et al., 2013; Schrödinger, 2023e) in Maestro (Schrödinger, 2023b). The System Builder tool was employed to prepare all systems, utilizing the OPLS-2005 (Bowers et al., 2006) force field with the TIP3P (Hornak et al., 2006) solvation model in an orthorhombic box. To achieve the physiological equilibrium, the systems were neutralized by introducing 0.15 M sodium chloride. The simulations were executed at 1 atm and 310.0 K using Martyna-Tobias-Klein barostat (Martyna et al., 1994) and Nose-Hoover thermostat in an NPT ensemble (Nosé, 1984; Hoover, 1985). Prior to the simulation, the models underwent a relaxation process, and the stability of these complexes was analyzed during a 100 ns MD production run. Trajectories were saved at 100 ps intervals, and the simulation’s stability was assessed by comparing the Nb-RBD’s root mean square deviation (RMSD) and root mean square fluctuation (RMSF) over time (Maiorov and Crippen, 1994).

### 2.8 MM/GBSA analysis

Following MD simulations, binding free energy (ΔG) calculations were performed at intervals of 20 ns using the Prime (Jacobson et al., 2002; 2004; Schrödinger, 2023d) on equilibrated MD trajectories to quantify the thermodynamic drivers of Nb-RBD interactions. The total ΔG was computed as:

$$\Delta G=G_{Complex}-\left(G_{RBD}+G_{Nb}\right)$$

where G_Complex_, G_RBD_, and G_Nb_ represent the free energies of the bound complex, isolated RBD, and isolated Nb, respectively. The MM/GBSA energy decomposition dissects ΔG into six key components:

ΔG_Coulomb_ quantifies electrostatic interactions between charged residues, calculated using Coulomb’s law, capturing the influence of charge−charge interactions on ΔG. ΔG_vdW_ encompasses van der Waals contributions, including both attractive dispersion forces and repulsive Pauli exclusion effects, reflecting short−range atomic interactions. ΔG_SolvGB_ quantifies polar solvation energy, modeled through the Generalized Born approximation to account for solvent−mediated electrostatic shielding and desolvation penalties. ΔG_Lipo_ measures hydrophobic burial energy, proportional to the reduction in solvent−accessible surface area (SASA) of non−polar residues, capturing the entropic gain from solvent exclusion. ΔG_Hbond_ denotes energy contributions from hydrogen bond formation, derived from geometric and electrostatic criteria, highlighting the role of specific polar interactions. Finally, ΔG_Covalent_ represents internal strain energy arising from bonded interactions (bonds, angles, dihedrals) within the Nb or RBD during binding, reflecting conformational changes induced by complex formation.

To account for entropic effects, a quasi−harmonic entropy analysis was integrated into the workflow. Conformational entropy changes (ΔSbinding) were computed from MD trajectories using Maestro’s Simulation Interaction Analysis tool (Schrödinger, 2023b), which employs a quasi−harmonic approximation to estimate vibrational entropy:

$${\Delta S}_{binding}=S_{complex}-\left(S_{RBD}+S_{Nb}\right)$$

The total binding free energy was then thermodynamically refined using:

$$\Delta G={\Delta G}_{MM/GBSA}-{T\Delta S}_{binding}$$

where T is the simulation temperature (300 K).

## 3 Results

### 3.1 RBD refinement and analysis

**FIGURE 2:**
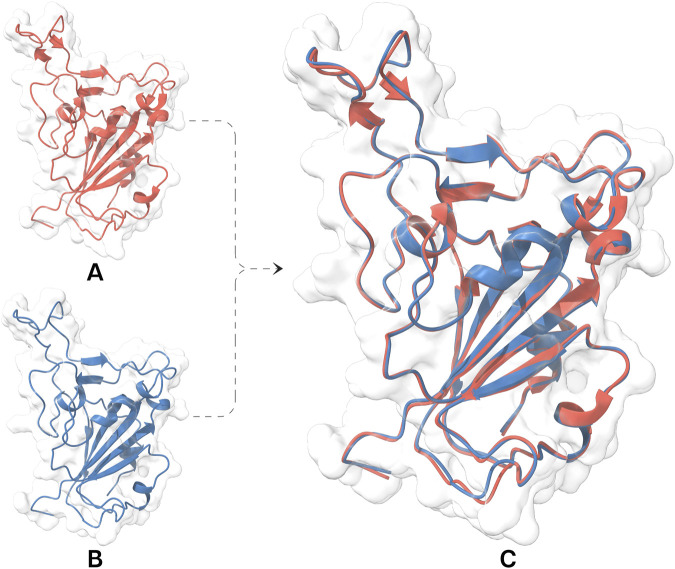
Structural representation and evaluation of the RBD facilitating ECSb nanobody construction. **(A)** 3D structure of RBD (red) retrieved from protein data bank underwent structural refinement protocols resulting in a refined RBD structure (blue; **(B)** with fewer structural issues. **(C)** A visual comparison of the two structures, highlighting the improvements achieved through the refinement process. Both models fitted well within electron density maps of 7VYR_R.

A thorough review using Protein Structure Reliability reports identified RBD (7VYR_R) as the most suitable candidate due to its minimal structural defects. This RBD structure was further refined by removing ions and crystallographic water molecules located beyond 5 Å, introducing disulfide bonds, and capping termini to get stable and correct conformation. Hydrogen bond networks were optimized through the prudently incorporation of hydrogens and the rotation of the amide (Asn, Gln, and His) and hydroxyl (Ser, Thr, and Tyr) functional groups. Energy minimization improved peptide planarity for specific bonds (C336−P337, E340−V341, T345−R346, N360−C361, and A388−D389) and reduced temperature factor for L518, H519, and A520. Loop optimization improved backbone dihedral angles (Φ and Ψ) for A372, K386, F392, D442, and Y505 (Supplementary Table S1). These refinements effectively addressed the structural issues present in RBD structure (Figure 2).

### 3.2 Epitopes characterization and electrostatic analysis

The analysis of titration curves over a pH range of 2–12 to determine the pKa values of ionizable residue in each epitope was conducted (Supplementary Figure S1). In addition, PROPKA [59–61] was used to visualize the pKa values of epitope residues at a pH of 7.2 ± 0.2 (Supplementary Table S2). The epitopes were characterized by their number of ionizable residues: CA1 had the most (9), followed by AS3 (6), AS1 (4), AS2 (2), and CA2 (2). This analysis suggested that CA1 would have the strongest and the most favorable EC, followed by AS3 and AS1.

Surface analyses assessed the overall electrostatic charge of the epitopes and RBD. CA1 and AS3 exhibited the highest positive charge, while AS1 demonstrated the most negative charge. These findings established the basis for recommendation for replacing specific charged amino acids in FRs with those selected for mutation from the prior structure.

### 3.3 ECSbs construction

**FIGURE 3:**
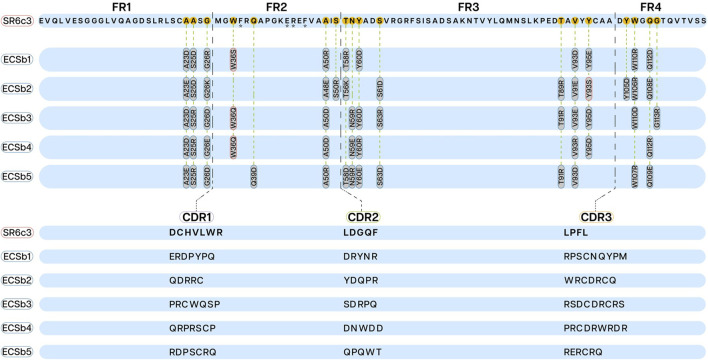
Framework regions (FRs; upper section) and complementarity−determining regions (CDRs; lower section) sequences displaying mutations in ECSbs sequences generated through integrated engineering strategy. The mutations introduced into the FRs (FR1, FR2, FR3, and FR4) of ECSbs sequences to minimize aggregation propensity are outlined in red and mutations to induce complementary dipole are outlined in blue. The CDR sequences of ECSb variants are aligned and compared with the SR6c3 reference sequence, illustrating the modifications made to optimize binding affinity and electrostatic potential. Asterisks under residues (F37, E44, R45, and V48) represent the VHH hallmark residues.

This study presents a novel approach to FR engineering, building upon the SR6c3 Nb for an integrated strategy (Chen X. et al., 2021). This approach uniquely integrates CDR design with the optimization of the overall electrostatic potential between the epitope and the RBD. Unlike traditional FR engineering approaches that primarily focus on stability, solubility, and immunogenicity (Lawrence et al., 2007; Miklos et al., 2012), this approach empowers FRs to actively contribute to enhanced binding affinity and specificity in conjunction with the CDRs. We strategically engineered specific amino acids in both the FRs and CDRs of the ECSb sequences to minimize aggregation propensity, induce complementary dipole, and optimize electrostatic potential (Figure 3; Supplementary Table S3). Balancing net charges of CDRs and entire ECSbs with their corresponding epitopes and RBD, led to improved ECSb binding to the RBD. To specifically isolate the effect of EC on binding affinity and specificity, a minimal number of hydrophobic residues were introduced, as described earlier (Chothia and Janin, 1975; Rose et al., 1985; Nicholls et al., 1991).

**FIGURE 4:**
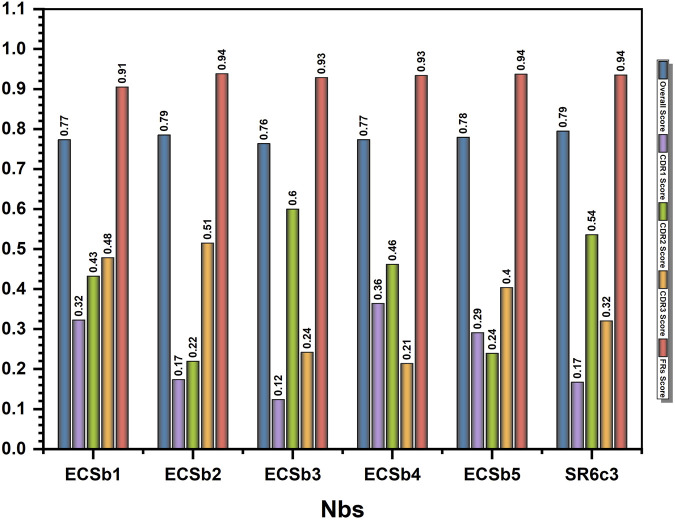
Humanization profile of nanobodies evaluated by AbNatiV. The bar chart comparing the computational humanization profile of the whole nanobodies (ECSbs and SR6c3) and their overall score, CDR score, and FR scores. The x−axis represents the names, and the y−axis represents the score.

All Nbs (ECSbs and SR6c3) demonstrated strong nativeness (overall) scores, with values consistently near the threshold level of 0.8, ranging from 0.76 to 0.79 (Figure 4). ECSb2 and SR6c3 exhibited the highest scores (0.79), while ECSb3 had a lower score (0.76). Notably, the FR sequences of all Nbs consistently demonstrated high nativeness, surpassing the threshold with an average score of 0.93, due to their high sequence conservation. In contrast, the CDRs exhibited significantly lower nativeness scores (<0.8), ranging from 0.12 to 0.6, reflecting their completely randomized sequences (Figure 4).

These results demonstrate the potential of our novel approach in Nb engineering, balancing EC with structural integrity. The high nativeness scores of the FRs, coupled with the strategically designed CDRs, suggest that our method successfully integrated FR engineering with CDR design while maintaining overall antibody structure.

### 3.4 Structure prediction analysis

All predicted structures of Nbs demonstrated high confidence, with pLDDT score consistently exceeding 85 (Supplementary Figure S2). ECSb2 demonstrated the highest average pLDDT scores of 93.6, followed by SR6c3 at 92.9, ECSb5 at 90.5, ECSb1 at 89.6, ECSb4 at 88.8, and ECSb3 at 87.8. Each Nb exhibited distinct variations in pLDDT scores, suggesting distinct structural characteristics within their flexible regions. Consistent with other protein structure prediction tools (Du et al., 2021; Su et al., 2021; Cohen et al., 2022; Lin et al., 2022; 2023; Wang W. et al., 2022; Wu et al., 2022; Ruffolo et al., 2023), pLDDT scores were generally lower in loop regions but remained high (>95) in the non−loop region (Supplementary Figure S2).

### 3.5 Structural refinement and analyses

Structural refinements of the Nbs were performed using Prime (Jacobson et al., 2002; 2004; Schrödinger, 2023d). However, ECSb3 and ECSb4 exhibited persistent structural anomalies, including distorted backbone and sidechain dihedrals, deviations in bond angles and lengths, non−planer peptides, and the buried unsatisfied hydrogen bond acceptors. These issues were rectified through additional energy minimization and loop refinement, though this process was challenging due to the exacerbating factors such as temperature, bond angle and length deviations, steric clashes, and sidechain dihedrals. The remaining Nb structures demonstrated satisfactory reliability, as validated by PSRs and Ramachandran Plots (Supplementary Table S4; Supplementary Figure S3).

Surface evaluations of the refined structures were conducted using BioLuminate (Beard et al., 2013; Salam et al., 2014; Zhu et al., 2014; Schrödinger, 2023a) to assess their electrostatic characteristics, aggregation tendency, and reactivity. Aggregation score calculations identified SR6c3 as having the highest aggregation propensity (190.548), followed by ECSb5 (70.451), while ECSb3 exhibited the lowest score (0.853) (Table 1). This observation implies that SR6c3 and ECSb5 have a higher propensity for aggregate formation and can lead to structural misfolding during *in vitro* or *in vivo* development compared to the remaining Nbs (Lijnzaad et al., 1996; Nissley et al., 2022; Van Gils et al., 2022; Peruzzi et al., 2024). Patch analysis further elucidated the basis of aggregation scores by examining the distribution, intensity, and orientation of hydrophobic patches (Sankar et al., 2018) (Supplementary Figure S4C; Supplementary Figure S5). SR6c3, with its 85% hydrophobic amino acids, displayed five hydrophobic patches with an average intensity of 0.639. In contrast, ECSb1 and ECSb3 had fewer patches, 6 and 4 with average intensity of 0.543 and 0.503, respectively. Regarding distribution and orientation, patches in SR6c3 are clustered majorly around CDRs, driven by the substantial involvement of hydrophobic residues, which facilitate orientation changes due to CDR flexibility. Conversely, ECSb1 and ECSb3 had highly scattered hydrophobic patches along FRs, hindering aggregate formation.

**TABLE 1:** Comparison of the sum of aggregation score (AggScore) and thermostability between ECSbs and SR6c3.

Nbs	Sum of AggScore	Thermostability (kcal.mol^−1^)
ECSb1	9.094	100.4
ECSb2	0.927	96.72
ECSb3	0.853	110.9
ECSb4	18.347	148.3
ECSb5	70.451	109.8
SR6c3	190.548	62.6

Our electrostatic engineering strategy increased the number and intensity of negatively charged patches on ECSbs, enhancing their solubility potential and reducing aggregation propensity (Kramer et al., 2012; Ganesan et al., 2016; Carballo-Amador et al., 2019). Notably, ECSbs exhibited an average of 12 patches with an intensity of 1.26, while SR6c3 exhibited 15 scattered patches with a lower intensity of 1.10 (Supplementary Figures S4A, B; Supplementary Figure S5). The higher average intensity of ECSbs is due to distribution of 64 residues on average in 12 patches compared to SR6c3’s 64 residues distribution in 15 patches (Supplementary Figure S6).

Assimilation of more charged residues; 13 residues in positive patches and 12 residues in negative and four residues in hydrophobic patches in ECSbs’ CDRs result in higher electrostatic steering potential than SR6c3 (Wade et al., 1998; Patil and Nakamura, 2007; Tsutakawa et al., 2017) (Supplementary Figure S4; Supplementary Figure S7). As in contrast, SR6c3 CDRs have 5 positively charged residues, 13 negatively charged, and 10 hydrophobic residues. This finding aligns with the above results of higher aggregation around CDRs in SR6c3 (Supplementary Figure S5; Supplementary Figure S6).

Our integrated approach successfully developed electrostatically complementary patches and potential between the Nbs and specific epitopes, as well as the overall RBD. ECSbs, such as ECSb1, exhibited a balance distribution of positively (16) and negatively charged (14) residues, complementing AS1’s net charge of ‒2e. Across the full ECSb1 structure, 59 residues contribute to positively charged patches and 67 to negatively charged patches, aligning with the overall RBD charge of +2e (Supplementary Figure S6). Similar pattern was consistent across all ECSbs.

BioLuminate (Beard et al., 2013; Salam et al., 2014; Zhu et al., 2014; Schrödinger, 2023a) was also employed to predict reactive hotspots in the Nbs structures, focusing on vulnerabilities to post−translational modifications (PTMs), including cysteine modifications, glycosylation, deamination, oxidation, proteolysis, and Asp isomerization (Farnsworth et al., 1989; Cohen, 2002; Blom et al., 2004; Ohtsubo and Marth, 2006; Aebersold et al., 2018; March and Smith, 2020). These PTMs can significantly impact protein stability, immunogenicity, and function (Tsien, 1998; Hermeling et al., 2004; Walsh, 2010; Kumar et al., 2016; Lee et al., 2023). While no N− or O−linked glycosylation sites were identified in any of the Nbs, several reactive PTM hotspot, including deamidation, oxidation, proteolysis, Aspartate isomerization, and free cysteine sites, were found, with SR6c3 exhibiting the highest number (38) and ECSb3 the fewest (25) (Supplementary Table S5). Proteolysis and oxidation were the predominant PTMs across all Nbs, with SR6c3 showing the highest number of oxidation hotspots (14), indicating a higher susceptibility to oxidative damage in comparison to the other Nbs. In contrast, ECSbs displayed a higher number of proteolytic sites, suggesting increased vulnerability to proteolytic degradation compared to SR6c3.

Thermostability predictions using FoldX (Schymkowitz et al., 2005) revealed significantly higher thermostability in the ECSbs compared to SR6c3, with ECSb4 demonstrating the highest thermostability at 148.3 kcal.mol^−1^, while SR6c3 had the lowest (62.6 kcal.mol^−1^) (Table 2). Further analysis of intramolecular interactions, including hydrogen bonds (HBs), salt bridges (SBs), repulsive ionic bonding (RIB), disulfide bonds (DiBs), π−π stacking (Pistacking), π−cation (PiCation) interactions, and hydrophobic interactions (HPh), revealed that ECSbs possess superior characteristics. Electrostatic engineering resulted in a greater number of SBs in ECSbs, which contain an average of seven SBs compared to four in SR6c3 (Supplementary Figure S7). Moreover, SR6c3 possesses four RIBs, while ECSbs typically have only one RIB per structure. SR6c3 also lacks both π−π stacking and π−cation interactions, which significantly contribute to changes in free energy (ΔΔG), another factor of its lower stability. Furthermore, the presence of more DiBs in ECSbs, as compared to SR6c3, enhances their overall stability. In terms of HBs, ECSb5 and ECSb2 exhibit the highest number of interactions, with 33 and 32 HBs respectively, surpassing SR6c3, which has 25 HBs (Supplementary Figure S7). In contrast, SR6c3, due to a greater number of hydrophobic residues, has a higher propensity for aggregation, resulting in 32 HPh interactions compared to an average of 28 HPh interactions in ECSbs.

**TABLE 2:** Comparison of the binding free energy (ΔG), Coulombic component of binding free energy (ΔGC), hydrogen bonding component of binding free energy (ΔGHB) between ECSbs and SR6c3.

Nbs	ΔG (kcal.mol^−1^)	ΔGC (kcal.mol^−1^)	ΔGHB (kcal.mol^−1^)
ECSb1-RBD	−88.911	14.966	−5.252
ECSb2-RBD	−71.777	105.049	−2.867
ECSb3-RBD	−57.452	43.808	−8.705
ECSb4-RBD	−65.942	56.526	−8.785
ECSb5-RBD	−90.094	121.156	−11.802
SR6c3-RBD	−70.525	−12.828	−8.421

**FIGURE 5:**
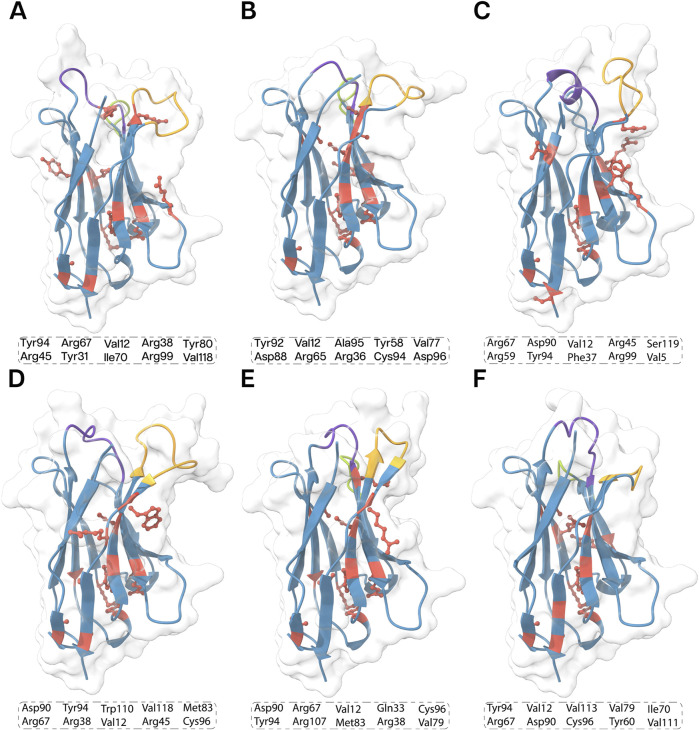
3D structures of the all the nanobodies, **(A)** ECSb1, **(B)** ECSb2, **(C)** ECSb3, **(D)** ECSb4, **(E)** ECSb5, and **(F)** SR6c3 highlighting key residues contributing to their stability. The top 10 critical residues for each nanobody are listed in the boxes. Structural regions are color−coded as follows: purple for CDR1, green for CDR2, orange for CDR3, and red for key stability residues. The background density maps provide a visual context for the Nb structure.

A total of 10 key residues contributing to Nb stability were identified due to their prominent involvement in the intramolecular interaction landscape (Figure 5). While the precise functions of these residues remain to be fully elucidated, their potential roles can be inferred. Cysteine residues likely stabilize the nanobody through disulfide bond formation. Arginine, a positively charged amino acid, may form salt bridges, contributing to stability and antigen binding. Aromatic residues like tyrosine can participate in various interactions, including pi−pi stacking and hydrogen bonding. Hydrophobic residues like valine often stabilize the protein’s core. However, further research is needed to fully understand the specific roles of these residues in nanobody function.

The integrated electrostatic engineering approach effectively increased the number of SBs in ECSbs. Notably, mutations such as S50R, S61D, and W106R in ECSb2; G26D, A50D, N59R, Y95D, and W110D in ECSb3; and A23E, S25R, Q39D, S63D, and W107R in ECSb5 were instrumental in SB formation. Conversely, the two RIB interactions present in SR6c3 (W105−R45 and Y95−R45) are attributed to residues W105 and Y95, which are mutated in ECSbs. The comparison of thermostability results with the intramolecular contact analysis of Nbs elucidated one of the contributing factors to the heightened stability in ECSbs compared to SR6c3 (Vogt et al., 1997; Kumar et al., 2000). These computational analyses indicated that the electrostatic integration strategy was successful in enhancing the thermostability in ECSbs compared to SR6c3.

### 3.6 Protein−Protein docking

MM/GBSA calculations was used to rank the 30 poses of each Nb-RBD docked system based on their binding free energy. Notably, ECSb5-RBD pose (P) 15 exhibited the highest binding free energy (ΔG) of 90.094 kcal.mol^−1^, closely followed by P26 of ECSb1-RBD at 88.911 kcal.mol^−1^. SR6c3-RBD ranked fourth with ΔG of 70.525 kcal.mol^−1^, while ECSb3-RBD P14 exhibited the lowest ΔG at 57.452 kcal.mol^−1^ (Table 2).

**FIGURE 6:**
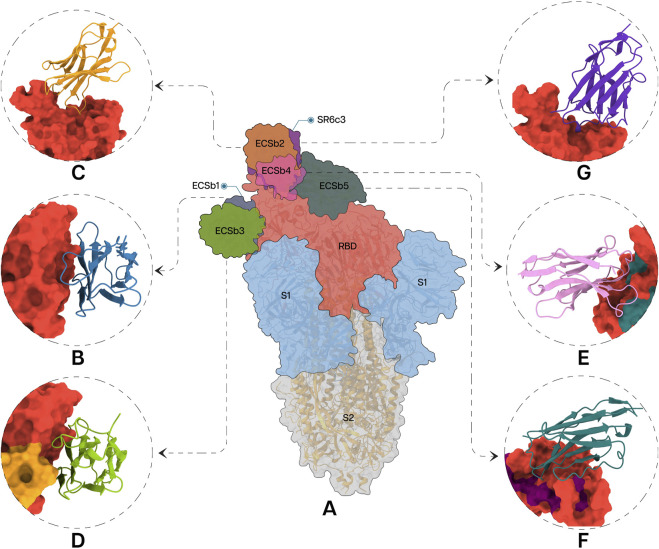
Specific interactions between RBD **(A)** and the Nbs; ECSb1 **(B)**, ECSb2 **(C)**, ECSb3 **(D)**, ECSb4 **(E)**, ECSb5 **(F)**, and SR6c3 **(G)** are highlighted in the circles.

Each Nb in the docked pose with the highest ΔG exhibited a distinct binding mode relative to the whole spike protein (Figure 6). ECSb1 and ECSb3 bound to a cryptic site within the RBD, whereas ECSb2, ECSb4, and SR6c3 targeted the receptor-binding motif (RBM) region. Detailed analysis of ECSb1 and ECSb2 interactions revealed unexpected binding patterns. Contrary to their intended targets (AS1 and AS2), these Nbs demonstrated a preference for residues that offered a more favourable binding architecture. This unexpected behaviour was characterized by the formation of two hydrogen bonds, resulting from engineering within the FRs. ECSb1 bound to AS3 instead of AS1, whereas ECSb2 bound with CA1 instead of AS2. In contrast, other ECSbs exhibited specific interactions that aligned with their target antigenic sites, highlighting the binding specificity conferred by electrostatic complementation (Figure 6).

Visualization of the electrostatic potential maps for the binding interfaces of each Nb-RBD complex corroborated the results of calculated EC scores (Supplementary Figure S8). The electrostatic maps of the ECSb2-RBD interface revealed similar charge distributions, leading to repulsive interactions that resulted in the lower EC score−. In contrast, ECSb1-RBD and ECSb5-RBD complexes displayed both high EC and Sc, resulting from strong molecular attractions. For the SR6c3-RBD complex, the electrostatic map of SR6c3 exhibited a high negative charge density in the upper region and a high positive charge density in the lower right region, whereas the RBD displayed a slight negative charge density in the upper portion and a high positive charge density in the lower right region (Supplementary Figure S8). This analogous charge distribution reduced the EC score for the SR6c3-RBD complex.

**FIGURE 7:**
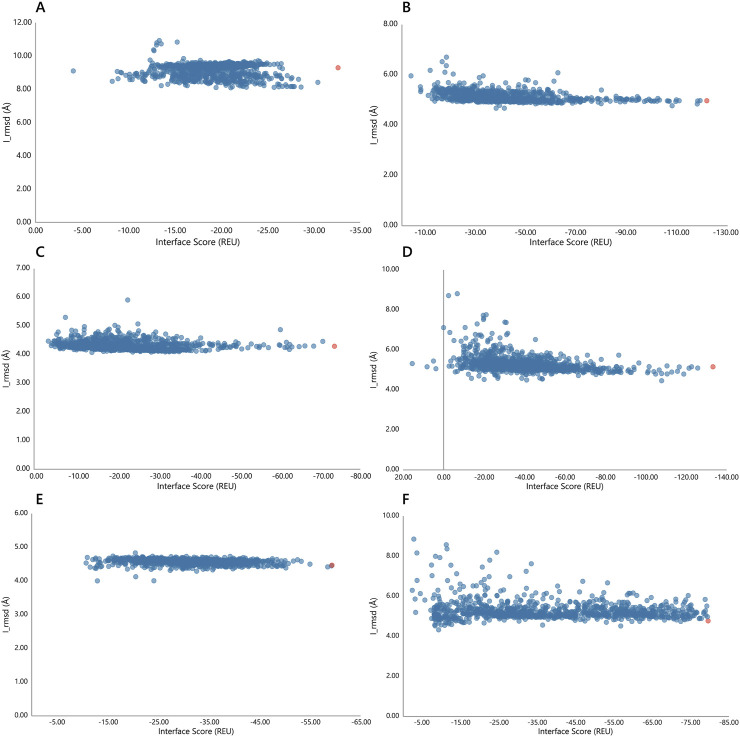
Docking funnels for each Nb-RBD complex. The interface scores (REU) are plotted against I_rmsd (Å) for each complex, **(A)** ECSb1-RBD, **(B)** ECSb2-RBD, **(C)** ECSb3-RBD, **(D)** ECSb4-RBD, **(E)** ECSb5-RBD, and **(F)** SR6c3-RBD. Red colored data points have the lowest interface scores.

In the second stage of docking, 1,000 decoys were generated for each complex using Rosetta, and the docking funnel was characterized by plotting interface scores (I_score, in Rosetta Energy Units, REU) against interface RMSD (I_rmsd). Among the engineered nanobodies, ECSb4-RBD exhibited the most favorable binding energy, with the lowest I_score of −133.232 REU, followed by ECSb2-RBD (−121.634 REU). In contrast, ECSb1-RBD showed the least favorable I_score (−32.619 REU) among the top-scoring decoys (Figure 7). The reference SR6c3-RBD demonstrated an intermediate I_score of −79.541 REU. For each complex, the decoy with the lowest I_score was extracted from the docking trajectory, representing the energetically optimal conformation for subsequent structural and functional analyses (Figure 9). These results highlight the enhanced interface energetics of ECSb4 and ECSb2 compared to both SR6c3 and other ECSb variants.

Among these structural and functional analyses, the comprehensive scoring elucidated the detailed energetic breakdown of Nb-RBD complexes. The major energetic contributions include Lennard-Jones attractive (LJ Att) and repulsive (LJ Rep) components, in conjunction with the Lazaridis-Karplus solvation energy (LK Sol) and Coulombic electrostatics (Elec), as well as hydrogen bond (HB) energies, collectively informed the calculation of the overall binding free energy (ddG). Among the complexes, ECSb1-RBD displayed the most favorable ddG of −802.83 REU, driven by robust LJ Att. of −1642.61 REU and HB energy of −280.36 REU (Table 3). Coupling with the highest dSASA 15,285.66 Å^2^, ECSb1--RBD’s scoring analysis yielded a normalized ddG/dSASA ratio of −0.0525. Conversely, although ECSb4-RBD was identified as possessing the lowest I_score (−133.232 REU) in the docking funnel, scoring analysis showed ddG (−592.35 REU) and dSASA (13,852.21 Å^2^) resulted in a less favorable normalized ddG/dSASA ratio of −0.04276 (Table 3). This lower ddG evidenced by higher LK Sol (973.46 REU) and LJ Rep (372.90 REU) coupled with lower individual energetic contributions of LJ Att; −1617.54 REU, Elec: −390.23 REU, and HB: −246.08 REU.

**TABLE 3:** Key scoring components including Lennard-Jones attractive (LJ Att), repulsive (LJ Rep), and solvation (LK Sol) energies, electrostatic contribution (Elec), hydrogen bonding (HB), and the resulting binding free energy change (ddG) per solvent−accessible surface area (dSASA) of Rosetta Energy Function 2015.

Complexes	LJ Att (REU)	LJ Rep (REU)	LK Sol (REU)	Elec (REU)	HB (REU)	ddG (REU)	dSASA (Å^2^)	ddG/dSASA
ECSb1-RBD	−1,642.61	341.93	956.97	−485.36	−280.36	−802.83	15,285.66	−0.053
ECSb2-RBD	−1,625.75	381.94	972.03	−425.82	−257.63	−613.41	13,995.39	−0.044
ECSb3-RBD	−1,655.05	356.30	997.17	−448.29	−277.92	−694.47	14,651.11	−0.047
ECSb4-RBD	−1,617.54	372.90	973.46	−390.23	−246.08	−592.35	13,852.21	−0.043
ECSb5-RBD	−1,680.14	395.94	975.82	−443.58	−288.20	−718.91	14,171.11	−0.051
SR6c3-RBD	−1,539.38	363.17	852.80	−358.00	−241.33	−615.84	15,117.49	−0.041

While ECSb5-RBD demonstrated robust binding energetics as well (ddG = −718.91 REU) yielding a normalized ddG/dSASA ratio of −0.0507 coupled with dSASA of 14,171.11 Å^2^ (Table 3). The second highest ddG was due to the major contributions of highest LJ Att (−1680.14 REU) and HB (−288.20 REU) energetics even with the highest LJ Rep of 395.94 REU and moderate score of LK Sol energy (975.81 REU). On the other hand, SR6c3-RBD showed intermediate ddG (−615.84 REU) even with lowest Coulombic (Elec: −357.99 REU) and hydrogen bonding (HB: −241.33 REU) energetics (Table 3). Although, it has lowest yet balanced LJ Att and LJ Rep −1539.38 REU and 363.17 REU, respectively. However, the lowest solvation penalty (LK Sol: 852.80 REU) and second highest buried surface area (15,117.49 Å^2^) improve ddG by requiring less energetic cost for desolvating the binding interface and increasing buried surface area to amplify the net favorable hydrophobic interactions. These data suggest that while Nb-RBD complexes may offer the most energetically favorable interactions, the interplay between individual energy components and the solvent exposure upon complex formation remains critical for the holistic assessment of binding affinity.

**FIGURE 8:**
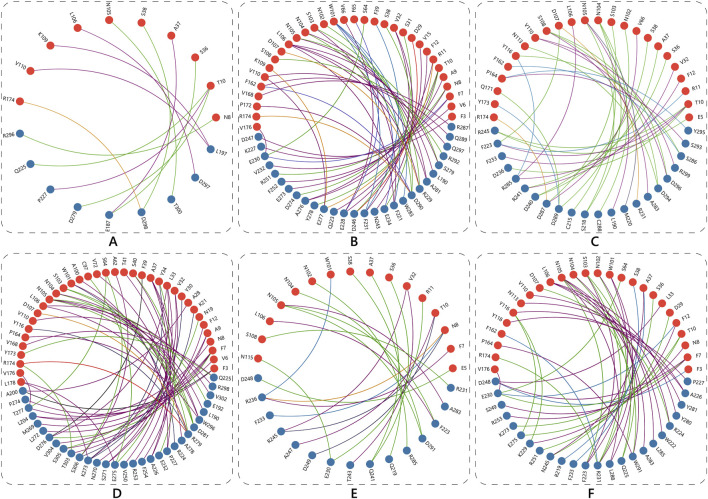
Schematic representation of interfacial interactions in the Nb-RBD complexes. The network of interactions for ECSb1-RBD **(A)**, ECSb2-RBD **(B)**, ECSb3-RBD **(C)**, ECSb4-RBD **(D)**, ECSb5-RBD **(E)**, and SR6c3-RBD **(F)**, with Nb residues shown as blue balls and RBD residues as red balls. The various types of interactions are color−coded as follows: Hydrophobic interactions (dark pink), π-Anion interactions (purple), Hydrogen bonds (green), π-π stacking (blue), Repulsive Ionic Bonding (red), Salt bridges (orange), and π-Cation interactions (grey).

The structure of interfacial interaction networks coupled with Sc and EC further characterized factors underneath these scorings. The ECSb-RBD complexes demonstrated distinct interfacial binding profiles compared to the SR6c3-RBD, as revealed by Sc and EC analyses. ECSb3-RBD achieved the highest Sc (0.7304), attributed to its extensive network of geometrically constrained interactions, including 17 hydrogen bonds (e.g., T10-D236) and five π-π stacking interactions (e.g., R11-F223) (Table 4; Figure 8). These interactions demand precise spatial alignment, enabling exceptional structural fit. In contrast, SR6c3-RBD exhibited moderate Sc (0.6302), limited by narrow interactions network (14 hydrogen bonds, five π−π) and the absence of salt bridges (Supplementary Table S6). Notably, ECSb1-RBD, despite its small interface (18 residues), displayed intermediate Sc (0.6851), supported by six hydrogen bonds and a stabilizing salt bridge (174R-298D), underscoring the impact of targeted polar interactions on structural alignment (Table 4; Figure 8).

**TABLE 4:** Interface characteristics including shape complementarity (Sc), electrostatic complementarity (EC) and number of interface residues for each Nb-RBD complex.

Complexes	Interface residues	Sc	EC
ECSb1-RBD	18	0.685	0.384
ECSb2-RBD	60	0.623	0.457
ECSb3-RBD	46	0.730	0.390
ECSb4-RBD	67	0.621	0.305
ECSb5-RBD	31	0.632	0.530
SR6c3-RBD	50	0.630	0.233

**FIGURE 9:**
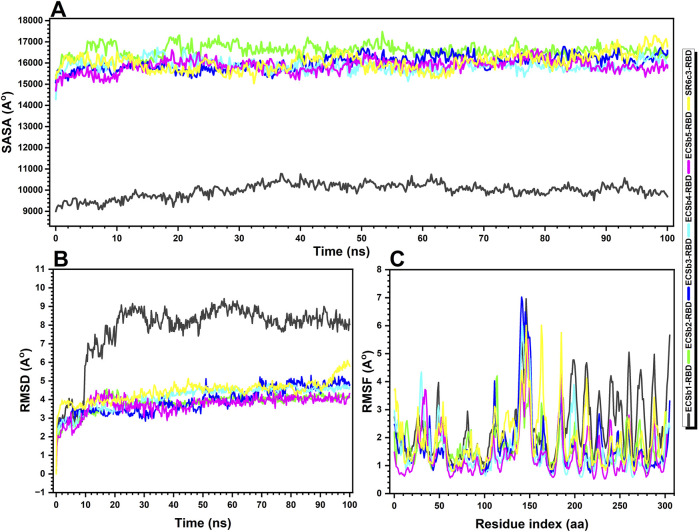
Structural and stability analysis of Nb-RBD complexes. **(A)** Solvent−accessible surface area (SASA) over time, highlighting the extent of exposure of hydrophobic and polar regions for the ECSb1-RBD (black), ECSb2-RBD (green), ECSb3-RBD (blue), ECSb4-RBD (cyan), ECSb5-RBD (purple), and SR6c3-RBD (yellow) complexes, **(B)** Root−mean−square deviation (RMSD) over time, reflecting the overall structural deviation during the simulations, and **(C)** Root−mean−square fluctuation (RMSF) analysis, indicating the flexibility of residues within each complex across the simulation, with higher RMSF values corresponding to greater flexibility. The data provide insights into the structural stability and conformational changes of the complexes throughout the simulation period.

Electrostatic optimization varied markedly across complexes. ECSb5-RBD exhibited the highest EC (0.5295), driven by strategic integration of salt bridges (N8−R236) and π-cation interactions (T10−D249), which enhanced charge complementarity despite its modest interface size (31 residues). Conversely, SR6c3-RBD showed the lowest EC (0.2325), a consequence of its reliance on non-polar interactions (28 hydrophobic, five π-π) and lack of salt bridges (Table 4; Figure 9). ECSb4-RBD, despite possessing the largest interface (67 residues), displayed poor EC (0.3047), as its interaction profile was dominated by hydrophobic clustering (39 instances), which lacks electrostatic specificity (Table 4).

The interplay between Sc and EC revealed design-driven trade-offs. While ECSb3-RBD achieved structural precision (Sc = 0.7304), its moderate EC (0.3901) suggested that geometric optimization alone insufficiently resolves electrostatic mismatches. In contrast, ECSb5-RBD prioritized charge complementarity, achieving superior EC (0.5295) through targeted electrostatic interactions, even with fewer total interactions (23 vs. SR6c3’s 47). Intermediate performers included ECSb2-RBD, with moderate EC (0.4570) linked to four salt bridges and three π-anion interactions, and ECSb4-RBD, whose low EC (0.3047) reflected over-reliance on hydrophobic contacts.

Collectively, ECSbs outperformed SR6c3-RBD in EC, with ECSb5-RBD achieving a 2.3-fold improvement. The SR6c3-RBD’s suboptimal EC underscored the limitations of natural systems in balancing hydrophobic packing with electrostatic optimization − a challenge mitigated in the ECSb series through rational design of interaction networks. These results highlight the efficacy of tailored electrostatic synergy (ECSb5) and structural precision (ECSb3) in advancing molecular recognition beyond natural benchmarks.

The comprehensive analysis of docking energetics, interface complementarity, and binding affinity reveals distinct mechanistic advantages and trade−offs among the ECSbs compared to the SR6c3. ECSb4-RBD, despite exhibiting the most favorable initial interface energy, demonstrated suboptimal ddG and EC. This disparity arises from its over-reliance on hydrophobic clustering (39 instances) and high solvation penalties, which offset favorable interfacial contacts. Conversely, ECSb1-RBD achieved the most favorable ddG, driven by robust LJ Att and HB, despite its small interface (18 residues). Its superior normalized ddG/dSASA ratio underscores efficient energy utilization per unit of buried surface area, highlighting the importance of packing density in hydrophobic−driven interactions.

ECSb3-RBD emerged as a paradigm of structural precision, with the highest Sc of 0.7304 due to its dense HB network (17) and π-π stacking (5). However, its moderate EC reflects unresolved electrostatic mismatches, illustrating that geometric optimization alone cannot fully compensate for charge misalignment. In contrast, ECSb5-RBD prioritized electrostatic synergy, achieving the highest EC through strategic salt bridges and π−cation interactions, which enabled a competitive ddG and normalized ratio despite fewer total interactions (23 vs. SR6c3’s 47). This underscores the efficacy of targeted charge complementarity in enhancing binding efficiency.

The SR6c3-RBD, while leveraging hydrophobic packing (28 instances) and moderate shape fit, exhibited the lowest EC and intermediate ddG. Its performance highlights inherent limitations in natural systems: a reliance on broad, spatially tolerant interactions limits electrostatic optimization, despite advantages in solvation cost and buried surface area.

### 3.7 MD simulations

The molecular dynamics (MD) simulations of Nb-RBD complexes revealed critical insights into their structural stability, interfacial dynamics, and solvent accessibility, elucidating the biophysical underpinnings of their binding efficacy. ECSb5-RBD emerged as the most stable complex, exhibiting the lowest average RMSD (3.72 Å) and RMSF (1.41 Å), indicative of a rigid, well−optimized interface (Figures 9B, C). This exceptional stability likely stems from its strategic integration of electrostatic interactions (e.g., salt bridges, π-cation pairs) and a high proportion of secondary structure elements (35.96% SSE, predominantly β-strands), which collectively minimize conformational entropy and resist structural deviations. The low RMSF, particularly at the binding interface, underscores restricted residue-level flexibility—a hallmark of geometrically precise and electrostatically complementary interactions, as previously identified in its high Sc and EC scores.

In stark contrast, ECSb1-RBD displayed the highest RMSD (7.74 Å) and RMSF (2.39 Å), reflecting significant conformational plasticity (Figure 9C). While this dynamic behavior suggests ongoing structural adjustments to optimize binding, the paradoxically low SASA (9,981.11 Å^2^) indicates a deeply buried interface dominated by hydrophobic clustering (Figure 9A). This duality implies that ECSb1-RBD’s binding mechanism prioritizes strong, enthalpically favorable van der Waals interactions and hydrogen bonds (HB: −280.36 REU) over rigid structural alignment, allowing it to maintain affinity despite flexibility (Table 3). However, the elevated solvation penalty (LK Sol: 973.46 REU) observed in prior scoring analyses likely exacerbates its conformational instability, as desolvation costs destabilize the complex over time.

ECSb2-RBD and ECSb3-RBD exhibited intermediate stability (RMSD: 3.81–3.94 Å) but distinct interfacial characteristics (Figure 9B). ECSb2-RBD’s high SASA (16,594.02 Å^2^) suggests a solvent−exposed interface, which may enhance adaptability through dynamic interactions but compromises hydrophobic stabilization. Conversely, ECSb3-RBD’s moderate SASA (15,998.14 Å^2^) aligns with its geometrically constrained network of hydrogen bonds and π-π stacks, balancing rigidity with partial solvent accessibility (Figure 9A). Notably, both variants lack the electrostatic optimization seen in ECSb5-RBD, underscoring the trade-off between structural precision and charge complementarity.

ECSb4-RBD, despite its expansive interface (67 residues), displayed suboptimal stability (RMSD: 4.13 Å) and moderate RMSF (1.59 Å), consistent with its over-reliance on hydrophobic clustering (39 instances) and poor EC (0.3047) (Figure 9). The lack of polar specificity likely permits minor interfacial fluctuations, as non-directional hydrophobic interactions fail to fully constrain residue mobility. This aligns with its elevated solvation penalties (LK Sol: 973.46 REU) and repulsive forces (LJ Rep: 372.90 REU), which collectively destabilize the complex despite favorable initial interfacial energy (Table 3).

The SR6c3-RBD exhibited intermediate metrics across all parameters (RMSD: 4.44 Å; RMSF: 1.94 Å; SASA: 16,048.02 Å^2^), reflecting its developmental honed balance of hydrophobic packing and moderate structural adaptability (Figures 9A–C). Its relatively low solvation penalty (LK Sol: 852.80 REU) highlights artificial selection’s preference for energetically efficient desolvation and broad interfacial contacts (Table 3). However, its limited EC and sparse salt bridges reveal inherent limitations in natural systems, which prioritize robustness over precision.

### 3.8 MM/GBSA analysis

The MM/GBSA methodology (Genheden and Ryde, 2015), applied to equilibrated MD trajectories, provides a comprehensive thermodynamic perspective on the binding free energies (ΔG) of ECSbs and SR6c3. This analysis reveals distinct binding mechanisms and highlights the potential of this EC based design of ECSbs.

ECSb1-RBD exhibits moderate binding affinity (ΔG: −51.25 kcal·mol^−1^), primarily driven by strong van der Waals interactions (ΔG_vdW_: −56.25 kcal·mol^−1^) and lipophilic stabilization (ΔG_Lipo_: −20.41 kcal·mol^−1^) (Table 5; Supplementary Table S7). These contributions reflect its deeply buried interface (low SASA: ∼9,981 Å^2^) and efficient hydrophobic packing density, which is further evidenced by transiently favorable ΔG values at 80 ns (−74.10 kcal·mol^−1^), correlating with maximal lipophilic burial (Supplementary Table S7). However, polar solvation penalties (ΔG_SolvGB_: +10.40 kcal·mol^−1^) and variable Coulombic contributions (ΔG_Coulomb_: +12.53 kcal.mol^−1^) partially offset these gains, aligning with its high desolvation costs (LK Sol: 973.46 REU) noted earlier (Supplementary Table S7; Table 3). The significant conformational flexibility observed in MD simulations (RMSD: 7.74 Å) suggests a substantial entropic penalty, which likely reduces binding affinity (Figure 10). Despite these trade−offs, ECSb1-RBD’s exceptional normalized energy density (ddG/dSASA: −0.0525) highlights its efficiency in leveraging hydrophobic-driven interactions, albeit at the expense of dynamic adaptability (Table 3).

**TABLE 5:** Binding free energies (kcal·mol⁻^1^) for each Nb-RBD complex, measured at 0, 20, 40, 60, 80, and 100 ns, along with the average (Avg) binding energy across the simulations.

Time (ns)	ECSb1-RBD (kcal.mol^−1^)	ECSb2-RBD (kcal.mol^−1^)	ECSb3-RBD (kcal.mol^−1^)	ECSb4-RBD (kcal.mol^−1^)	ECSb5-RBD (kcal.mol^−1^)	SR6c3-RBD (kcal.mol^−1^)
0	−55.02	−252.39	−233.05	−194.32	−118.03	−219.02
20	−27.85	−131.24	−165.99	−225.88	−95.56	−123.11
40	−55.63	−225.32	−105.19	−162.16	58.19	−29.40
60	−67.42	516.49	−115.79	−179.23	−78.33	−87.77
80	−74.10	−156.80	−49.45	−165.13	−110.61	−118.25
100	−27.46	−152.41	−44.93	−168.75	−42.01	−55.43
Avg	−51.25	−66.94	−119.07	−182.58	−64.39	−105.50

In similar, ECSb5-RBD achieves a moderate average ΔG (−64.39 kcal.mol^−1^), underpinned by ΔG_vdW_ of −93.95 kcal·mol^−1^ and ΔG_Lipo_ of −50.31 kcal.mol^−1^ contributions (Table 5; Supplementary Table S7). Its structural rigidity (RMSD: 3.72 Å) minimizes entropic penalties, allowing robust hydrophobic network−powered enthalpic gains to dominate. However, polar solvation (ΔG_SolvGB_: +24.33 kcal·mol^−1^) and electrostatic interactions (ΔG_Coulomb_: +16.46 kcal.mol^−1^) despite highest EC (0.5295) driven by salt bridges and π-cation pairs remains unfavorable (Supplementary Table S7). Notably, its low solvation penalties and high secondary structure content (SSE: 35.96%) minimized entropic costs, reflecting its rigid, pre-organized binding interface. This enthalpic precision underscores the success of rational design in optimizing electrostatic synergy.

ECSb4-RBD yielded highest ΔG (−182.58 kcal.mol^−1^), yet the decomposition of energy terms reveals critical weaknesses (Table 5). The Coulombic contribution (ΔG_Coulomb_: +49.02 kcal.mol^−1^) is destabilizing, aligning with the low EC (0.3047) and underscoring poor electrostatic optimization evidenced by interfacial repulsion (R174-R279 charge clash) (Supplementary Table S7; Table 4). Conversely, van der Waals (ΔG_vdW_: −116.65 kcal.mol^−1^) and lipophilic (ΔG_Lipo_: −28.85 kcal·mol^−1^) terms dominate the stabilization, consistent with the hydrophobic−driven interface (39 instances). However, the solvation energetics (ΔG_SolvGB_: −29.15 kcal.mol^−1^) and negligible hydrogen−bond contributions (ΔG_Hbond_: −0.92 kcal.mol^−1^) further highlight weak polar specificity (Supplementary Table S7). Temporal fluctuations in ΔG (e.g., ΔG_Coulomb_ ranging from −11.72 to +225.25 kcal.mol^−1^ over 100 ns) reflect transient electrostatic repulsions and interfacial rearrangements, indicative of an electrostatic instability. Contrarily, the progressive decline in covalent binding energy (ΔG_Covalent_) from −13.22 to −106.33 kcal·mol^−1^ suggests strengthening interfacial cohesion over time (Supplementary Table S7). This analogous inclination between ΔG_Coulomb_ and ΔG_Covalent_ over simulation trajectory reflects the influence of electrostatic instability on ΔG_Covalent_. However, the conversed drift in dominate energetics of ΔG_vdW_ (−138.66 to −59.38) and ΔG_Lipo_ (−81.03 to +20.97 kcal.mol^−1^) align with MD observations of rising RMSD (1.96 Å at 0.2 ns to 4.66 Å at 100 ns) (Supplementary Table S7; Figure 10).

ECSb2-RBD and ECSb3-RBD exhibited divergent profiles, reflecting their distinct interfacial strategies. ECSb2-RBD’s average ΔG (−66.94 kcal.mol^−1^) is driven by strong van der Waals (ΔG_vdW_: −114.96 kcal.mol^−1^) and lipophilic contributions (ΔG_Lipo_: −97.91 kcal.mol^−1^) (Table 5; Supplementary Table S7). At 60 ns of ΔG calculations trajectory, ECSb2-RBD displayed a pronounced outlier of +516.50 kcal.mol^−1^ driven by anomalously high ΔG_Covalent_, ΔG_Coulomb_, and ΔG_vdW_ (+103.81 kcal.mol^−1^, +207.60 kcal.mol^−1^, and +331.74 kcal.mol^−1^, respectively) contributions (Table 5; Supplementary Table S7). This likely reflects transient conformational artifacts or force field limitations. Excluding this outlier, the complex showed high ΔG (−183.63 kcal.mol^−1^), supported by robust ΔG_vdW_ (−204.30 kcal.mol^−1^) and ΔG_Lipo_ (−100.47 kcal.mol^−1^) interactions. However, its elevated SASA (∼16,594 Å^2^) incurred solvation penalties (ΔG_SolvGB_: + 17.63 kcal·mol^−1^), while reduced EC (0.4570) limited ΔG_Coulomb_ gains. This aligns with its intermediate stability (RMSD: ∼3.81 Å) and suggests a design compromise between adaptability and interfacial specificity. In contrast, ECSb3-RBD achieves the second highest average ΔG (−119.07 kcal·mol^−1^), supported by geometric precision (Sc: 0.7304) via extensive hydrophobic bonding (ΔG_vdW_: −129.73 kcal·mol^−1^) and π-π stacking (ΔG_Lipo_: −47.67 kcal.mol^−1^). However, its electrostatic inefficiency (EC: 0.3901) manifested in suboptimal Coulombic contributions (ΔG_Coulomb_: +17.08 kcal.mol^−1^). Although its moderate entropy and solvation penalty (ΔG_SolvGB_: +47.86 kcal.mol^−1^) tempers affinity, ECSb3-RBD’s structural precision exemplifies the advantages of engineered hydrogen bond networks in enhancing binding efficacy.

Conversely, SR6c3-RBD showed stronger ΔG (−105.50 kcal.mol^−1^), driven by favorable vdW (ΔG_vdW_: −80.58 kcal.mol^−1^) and lipophilic terms (ΔG_Lipo_: −56.59 kcal.mol^−1^) (Table 5; Supplementary Table S7). ΔG_vdW_ and ΔG_Lipo_ partially offset by weak electrostatic contributions (ΔG_Coulomb_: +175.61 kcal.mol^−1^) reflecting the lowest EC (0.3047), which were frequently counteracted by polar desolvation (ΔG_SolvGB_: −96.37 kcal.mol^−1^) (Table 5; Supplementary Table S7). These calculation trajectories aligned with the development prioritization of hydrophobic packing over charge optimization, a limitation that ECSbs effectively addresses. The highest electrostatic penalty and entropic costs reflect SR6c3-RBD’s natural trade−off between adaptability and stability, further underscoring the advantages of rational design in achieving superior binding profiles.

## 4 Discussion

This study aimed to explore the potential of electrostatically tailored Nbs as a therapeutic strategy against SARS-CoV-2, focusing on generating high-affinity and stable binding with RBD of the spike protein. Our findings demonstrated the successful development and characterization of ECSbs designed to target the Spike protein’s RBD. By leveraging computational biology and engineering, we created Nbs with enhanced electrostatic profile and binding affinities (Cheloha et al., 2020).

The structural refinement of the RBD effectively addressed various anomalies inherent in the initial model, enhancing its overall accuracy and stability. Disulfide bond formation,− the introduction of hydrogen bonds, loop refinements and energy minimizations of selected residues were pivotal in achieving conformational stability. Key residues (such as C336−P337, T345−R346, and A388−D389) demonstrated improved peptide planarity and reduced thermal fluctuations, particularly around the critical loop regions (A372, K386, and Y505). A prior study has shown that different amino acid residues R403, K/N/T417, L455, F486, Y489, F495, Y501, and Y505 on RBD play a key role in the protein recognition mechanism (Biskupek and Gieldon, 2024). Our methodical approach to ECSb design, focusing on optimizing electrostatic interactions through strategic amino acid modifications, proved effective in enhancing binding affinity and specificity towards the RBD (Khan et al., 2021). This aligns with the previous study (Zhu et al., 2023) that successfully engineered Nbs with higher binding affinity to the S protein using a combination of template selection, mutation analysis, and single−site saturated mutagenesis in CDRs. However, our methodical approach for ECSbs represents a novel approach in the field of Nb engineering and differ principally, as our study focused on ECSb design, through strategic amino acid modification both in CDRs and FRs based on electrostatic complementation (Entzminger et al., 2017; Gąciarz and Ruddock, 2017).

The engineered Nbs in this study exhibited varying binding patterns to the SARS-CoV-2 spike protein. Three of the five Nbs (ECSb3-ECSb5) successfully bound to the targeted antigenic sites, demonstrating their specificity. However, ECSb1 and ECSb2 displayed unexpected binding behavior, binding to alternative sites on the spike protein. ECSb1 bound to AS3 instead of the intended AS1, while ECSb2 bound to CA1 rather than AS2. These findings deviate from recent studies that have shown antibodies binding to the conserved fusion peptide region adjacent to the AS2 epitope (Premkumar et al., 2020; Dacon et al., 2022). The steric constraints imposed by the high density of spike proteins on the viral surface make this region difficult to access for antibodies (Low et al., 2022; Zhu et al., 2023). The unexpected binding patterns observed in ECSb1 and ECSb2 suggest that these Nbs may have evolved to overcome steric hindrances or target alternative regions on the spike protein.

The detailed features of the designed Nbs are presented in the supplemental material, providing a better comprehension of the biophysical principles underlying their binding efficiency. From the pKa−mapped ionizable residues essential for contact specificity (Supplementary Figure S1) to the structural predictions and validations that give our designed structures confidence (Supplementary Figures S2–S4). Supplementary Figures S1–S9 offer a visual compendium that captures everything. Interestingly, the surface patch analyses (Supplementary Figures S4–S6) point to possible aggregation sites, which is an important factor to consider during antibody design. This study demonstrates that our integration strategy simultaneously enhances binding affinity via EC optimization and improves aggregation propensity and thermostability, which should be considered when designing such candidates (Chiu et al., 2019; Muyldermans, 2021).

Electrostatic attraction plays a key factor in antibody-antigen interactions, enhancing binding specificity. Analysis of RBD epitopes based on their ionizable residues identified CA1 as the most favorable epitope for EC. Surface charge analysis of both Nb-RBD complexes and their binding interfaces reinforced the electrostatic differences between epitopes, with CA1 and AS3 being predominantly positively charged, while AS1 was more negative charged. These differences guided epitope-focused Nb design and mutation strategies to improve binding affinity. Engineered CDRs of ECSbs, paired with optimized FRs, enhanced EC, leading to higher binding specificity and stability (Tu et al., 2016). ECSbs demonstrated a better balance between net charge and electrostatic interactions, resulting in improved binding affinity to the RBD. This balance was particularly evident in ECSb2 and ECSb5, which exhibited high native structure scores and outperformed SR6c3 in terms of EC and CDR nativeness. The same idea could be implemented in bispecific antibody design, potentially resulting in more effective and scalable treatments (Fennell et al., 2013).

Rosetta docking, scoring, MD simulations, and MM/GBSA calculations revealed distinct mechanistic advantages and trade-offs for engineered nanobodies (ECSbs) compared to SR6c3. ECSb4-RBD, despite an initially favorable interface energy, exhibited suboptimal ddG and poor EC due to excessive hydrophobic clustering (39 instances) and high solvation penalties. This instability was further corroborated by MD simulations, which revealed moderate RMSF and RMSD values, indicative of limited polar specificity and ongoing interfacial fluctuations (Mangat et al., 2022). Conversely, ECSb3-RBD, with the highest Sc, demonstrated structural precision driven by extensive hydrogen bonding (17 H-bonds) and π-π stacking. While its MM/GBSA ΔG was high, suboptimal Coulombic contributions limited EC. Despite a well-packed interface, moderate EC and a positive ΔG_Coulomb_ suggest unresolved charge misalignments limit binding stability, consistent with previous findings (Zhou and Pang, 2018). ECSb2-RBD showed intermediate performance with a broad SASA, indicating a solvent-exposed interface that enhanced conformational adaptability but reduced hydrophobic stabilization, as seen in its moderate EC and Sc. A transient MM/GBSA outlier, likely from interfacial disruptions, highlighted the flexibility-stability trade-off (Childers and Daggett, 2017). Excluding this outlier revealed a more favorable ΔG, indicating hydrophobic and lipophilic interactions primarily drove binding. ECSb5-RBD’s balance of rigidity (low RMSD/RMSF) and electrostatic optimization (high EC, salt bridges, π-cation interactions) yielded a competitive MM/GBSA ΔG and impressive ddG/dSASA, highlighting the efficacy of targeted electrostatic design for enhancing binding efficiency, even with a moderate interface (Zhang et al., 2011). ECSb1-RBD, relying on a deeply buried hydrophobic interface, exhibited the most favorable ddG but high solvation penalties and entropic costs. SR6c3, in contrast, showed limitations in electrostatic optimization, with moderate ΔG, RMSD, and RMSF, underscoring the advantage of rational design (Derat and Kamerlin, 2022). These analyses demonstrate that ECSbs achieved improved binding affinity through tailored interfacial optimization, highlighting the importance of balancing shape complementarity, electrostatic interactions, and hydrophobic packing for potent nanobody design.

Our approach, which is based on in-depth structural and biophysical investigations, allowed us to precisely tailor Nbs to match the electrostatic environment of the RBD. Specifically, the ECSb1-RBD complex demonstrated a consistent interaction profile across simulated parameters, suggesting a viable treatment target. The dynamic nature of the interactions between ECSb5 and RBD, along with the varying surface area exposure, highlights the ability of modified Nbs to adjust to changing viral structures (Mei et al., 2022; Shi et al., 2022). The higher stability and affinity seen in ECSb1-RBD and ECSb5-RBD complexes are fundamentally derived from the harmonious interplay between surface and electrostatic compatibility at the binding interfaces, as illustrated by the docking poses and complementarity plots (Supplementary Figure S8). This additional data not only supports the conclusions made in the main text but also offers a concrete tool for research into Nb design in the future (Price et al., 2018).

*In silico* techniques have revolutionized antibody development, making significant contributions across various stages of the process. These approaches have been instrumental in several key areas: identifying antigen epitopes (Chen et al., 2019; Amitai, 2021; Mason et al., 2021; Tubiana et al., 2022; Xu et al., 2022), exploring the vast sequence space of CDRs (Lim et al., 2022; Pooja Mahajan et al., 2022; Prihoda et al., 2022), optimizing CDR sequences, predicting antibody structures (Ruffolo et al., 2023), forecasting binding modes (Schneider et al., 2022), and estimating the binding affinity between antibodies and antigens (Myung et al., 2022). These computational approaches have been particularly valuable in the context of SARS-CoV-2, accelerating the discovery and optimization of therapeutic antibodies (Taft et al., 2021; 2022; Shan et al., 2022; Shaver et al., 2022; Zhang et al., 2022). These advancements are enhancing our ability to design antibodies with improved binding affinity, specificity, and other desirable properties. Our work significantly contributes to the growing body of knowledge in Nb development by providing a comprehensive dataset that correlates residue substitutions in Nb CDRs and their binding affinity to the SARS-CoV-2 spike protein. This dataset not only adds to our understanding of Nb-RBD interactions, but also to support the future *in silico* driven Nbs engineering efforts. Such engineered Nbs have potential beyond binding affinity. They can be used as scaffolds for targeted drug delivery, conjugated with therapeutic agents or imaging probes (Sakthikumar et al., 2022). Studying Nb-viral protein interactions helps understand resistance mechanisms, informing strategies against emerging variants (Nasution et al., 2018). This multifaceted approach enriches our comprehension of Nb functionality and paves the way for innovative solutions in combating infectious diseases. By leveraging computational technologies, our study can advance the development of potent and targeted Nb−based therapeutics to combat SARS-CoV-2 and other pathogens.

## 5 Conclusion

Conclusively, our innovative and integrated computational approach to Nb engineering has yielded promising results, with ECSbs demonstrating superior stability, binding affinity, and EC compared to the referenced SR6c3. Notably, ECSbs exhibited significantly higher thermostabilities (100.4–148.3 kcal·mol⁻^1^) compared to SR6c3 (62.6 kcal·mol⁻^1^). In terms of binding free energy (ΔG), ECSb4-RBD and ECSb3-RBD achieved superior values of −182.58 and −119.07 kcal·mol⁻^1^, respectively, *versus* −105.50 kcal·mol⁻^1^ for SR6c3-RBD. Additionally, ECSb4-RBD and ECSb3-RBD showed enhanced electrostatic complementarity (EC) values of 0.305 and 0.390, respectively, compared to 0.233 for SR6c3-RBD. These key findings make a strong case for the incorporation of electrostatic principles into Nb design, indicating a significant improvement in our ability to combat viral infections through creative biotechnological developments. further experimental validation and optimization of the ECSb design process are a prerequisite.

## Data Availability

The original contributions presented in the study are included in the article/supplementary material, further inquiries can be directed to the corresponding authors.
