# Supplementary material for: Computational electrostatic engineering of nanobodies for enhanced SARS−CoV−2 receptor binding domain recognition
Source: Front Mol Biosci. 2025 Mar 10;12:1512788. doi: 10.3389/fmolb.2025.1512788 (PMC11931142; doi:10.3389/fmolb.2025.1512788)
Supplement: Supplementary file 1 [file DataSheet1.docx]

Supplementary Material

Computational Electrostatic Engineering of Nanobodies for Enhanced SARS-CoV-2 Spike Protein Recognition

**Zafar Iqbal^1†*^, Muhammad Asim^2 †^, Umair Ahmad Khan^3^, Neelam Sultan^4^, Irfan Ali^2*^**

^1^Central Laboratories, King Faisal University, Al-Ahsa P.O. Box 31982, Saudi Arabia. [zafar@kfu.edu.sa](mailto:zafar@kfu.edu.sa)

^2^Centre of Agricultural Biochemistry and Biotechnology (CABB), University of Agriculture, Faisalabad; Pakistan. [2019ag7915@uaf.edu.pk](mailto:2019ag7915@uaf.edu.pk) (M.A); [irfan.cabb@uaf.edu.pk](mailto:irfan.cabb@uaf.edu.pk) (I.A).

^3^Faisalabad Medical University, Medicine and Allied Department, Faisalabad, Pakistan. [umairwork.98@gmail.com](mailto:umairwork.98@gmail.com)

^4^Department of Biochemistry, Government College University Faisalabad, Pakistan. [biochemistrygcuf@gmail.com](mailto:biochemistrygcuf@gmail.com)

*** Correspondence:**Zafar Iqbal; Irfan Ali
[zafar@kfu.edu.sa](mailto:zafar@kfu.edu.sa) (Z.I); irfan.cabb@uaf.edu.pk (I.A).

^†^ These authors contributed equally to this work.


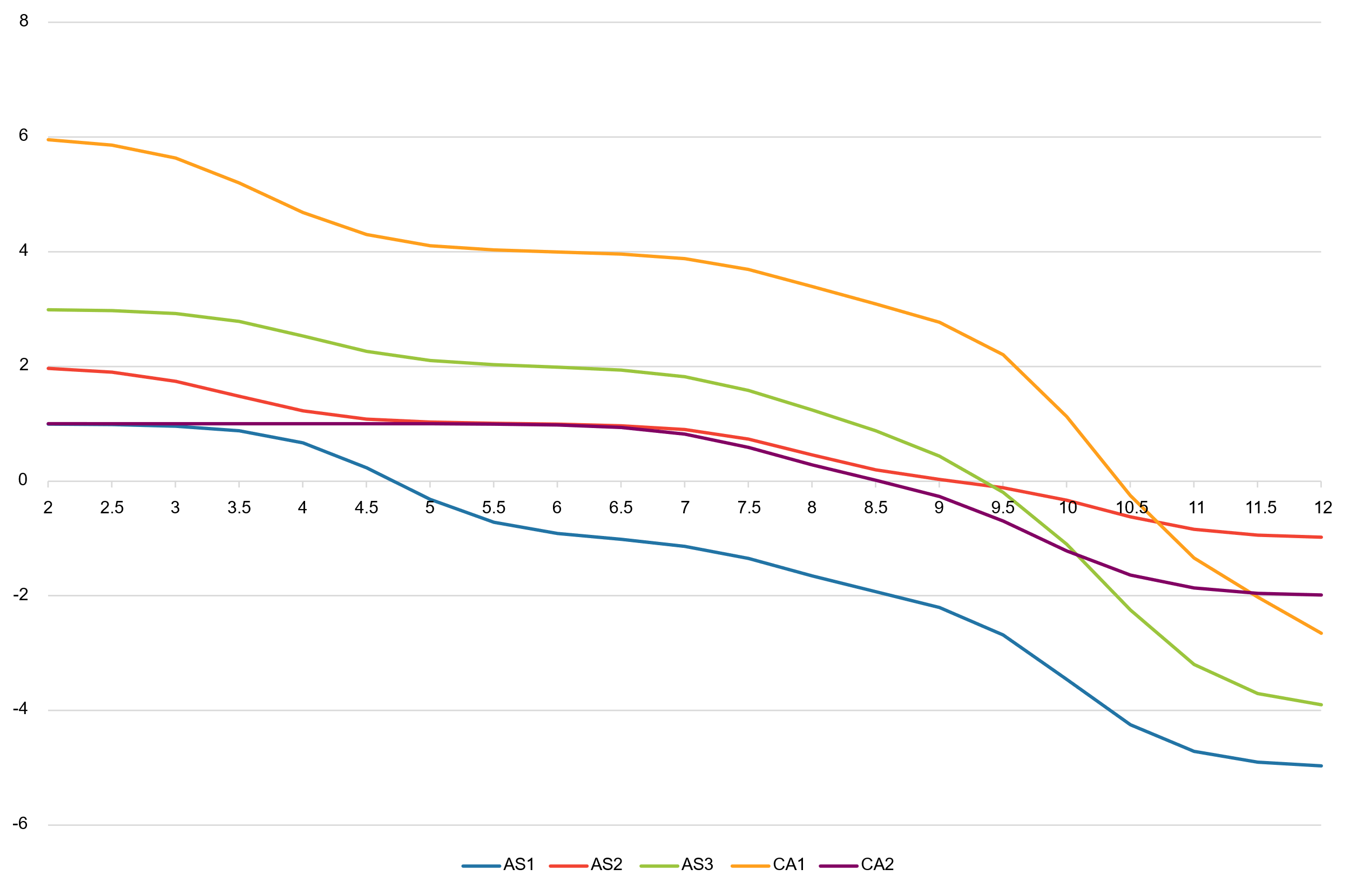


Figure S1. Titration curves of AS1, AS2, AS3, CA1, and CA2. Titration curves of each epitope showing specific charge at a range of pH from 2-12.


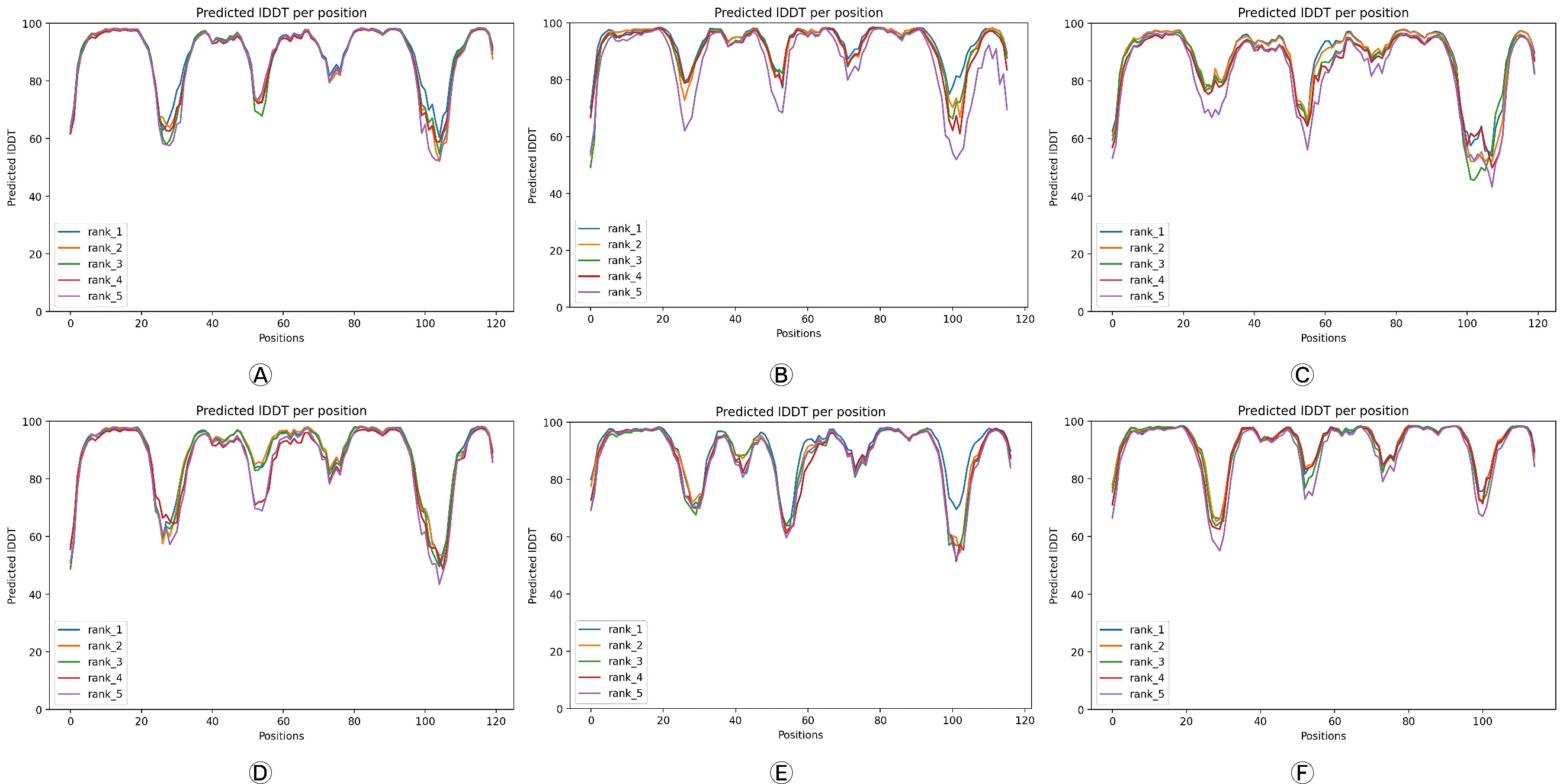


Figure S2. Structure prediction confidence for predicted nanobodies at each position. Per position local distance difference test (pLDDT) scores of the predicted structures of nanobodies A) ECSb1, B) ECSb2, C) ECSb3, D) ECSb4, E) ECSb5, and F) SR6c3 with rank 1-5 by prediction confidence.


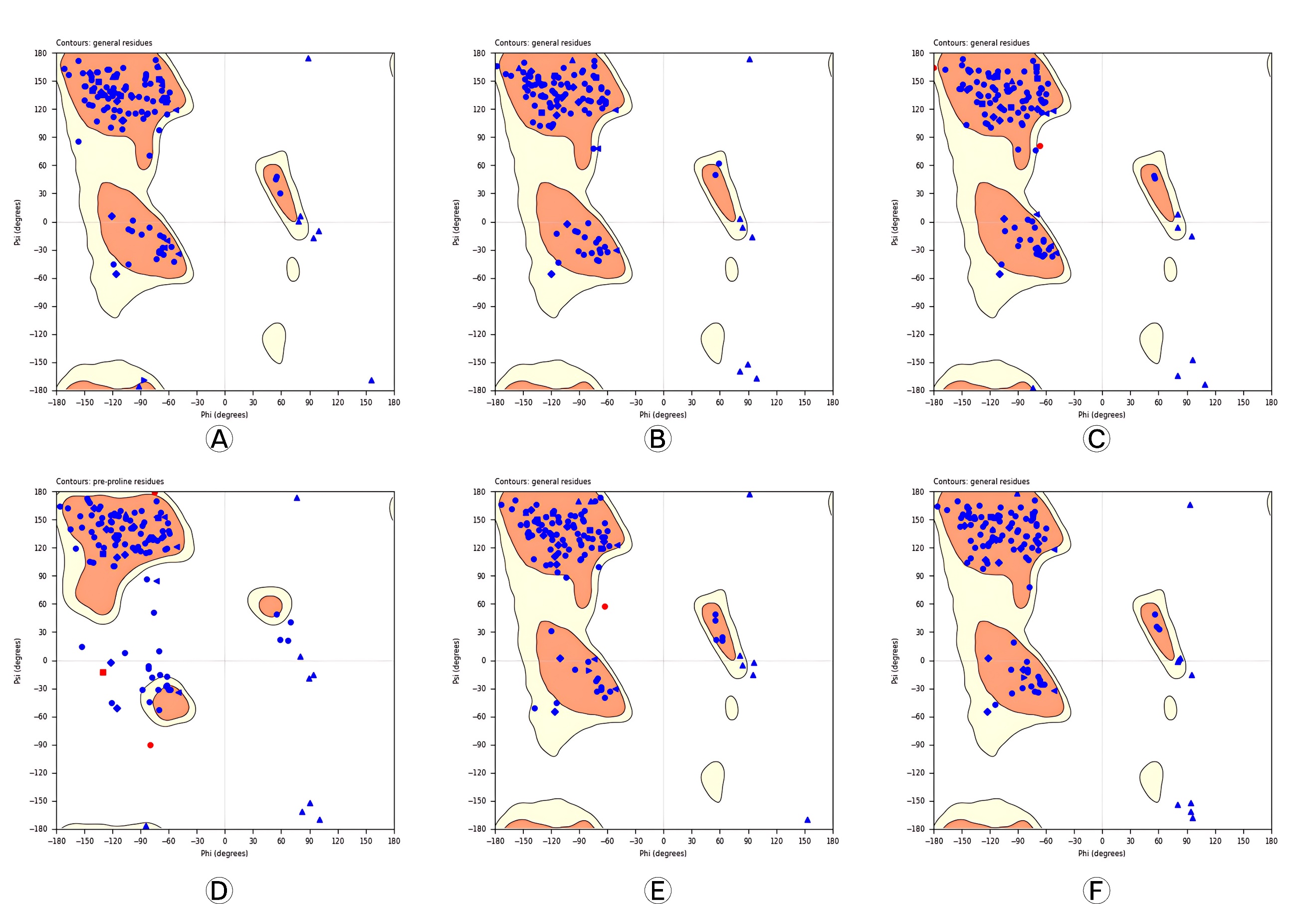


Figure S3. Structural validation of nanobodies through Ramachandran plots. Ramachandran plots representing structural quality of nanobodies A) ECSb1, B) ECSb2, C) ECSb3, D) ECSb4, E) ECSb5, and F) SR6c3 after preprocessing, energy minimizations, and loop refinements (ECSb3 and ECSb4).


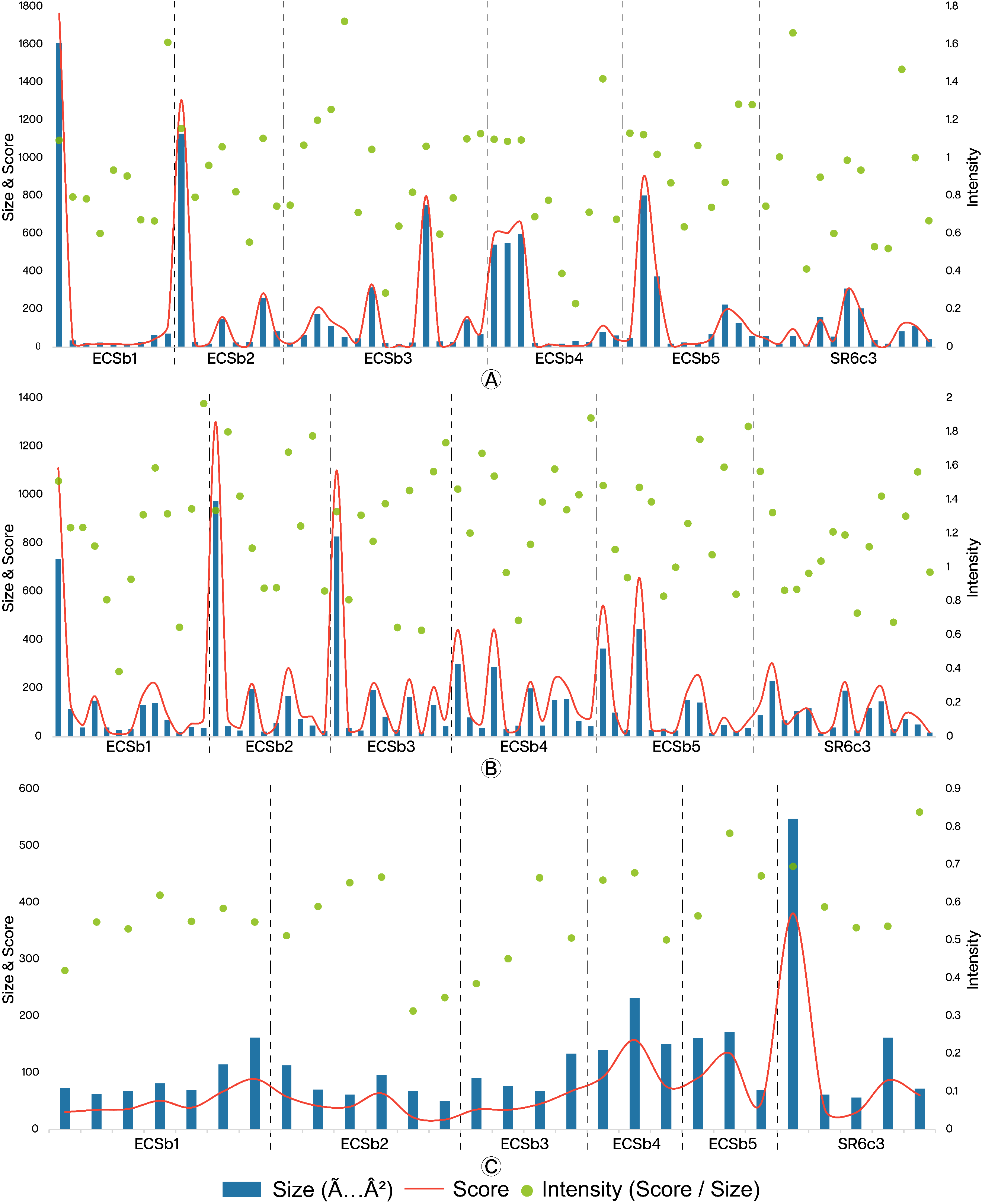


Figure S4. Patches of nanobodies: size, score, and intensity. A) Positively charged patches, B) Negatively charged patches, and C) Hydrophobic patches are represented. The distribution of positively charged patches in nanobodies (ECSbs and SR6c3), highlighting their Size (Å²), Score, and Intensity (calculated as Score/Size). The green bars represent the patch sizes, the red line traces the scores, and the blue dots indicate the intensity values for each patch.


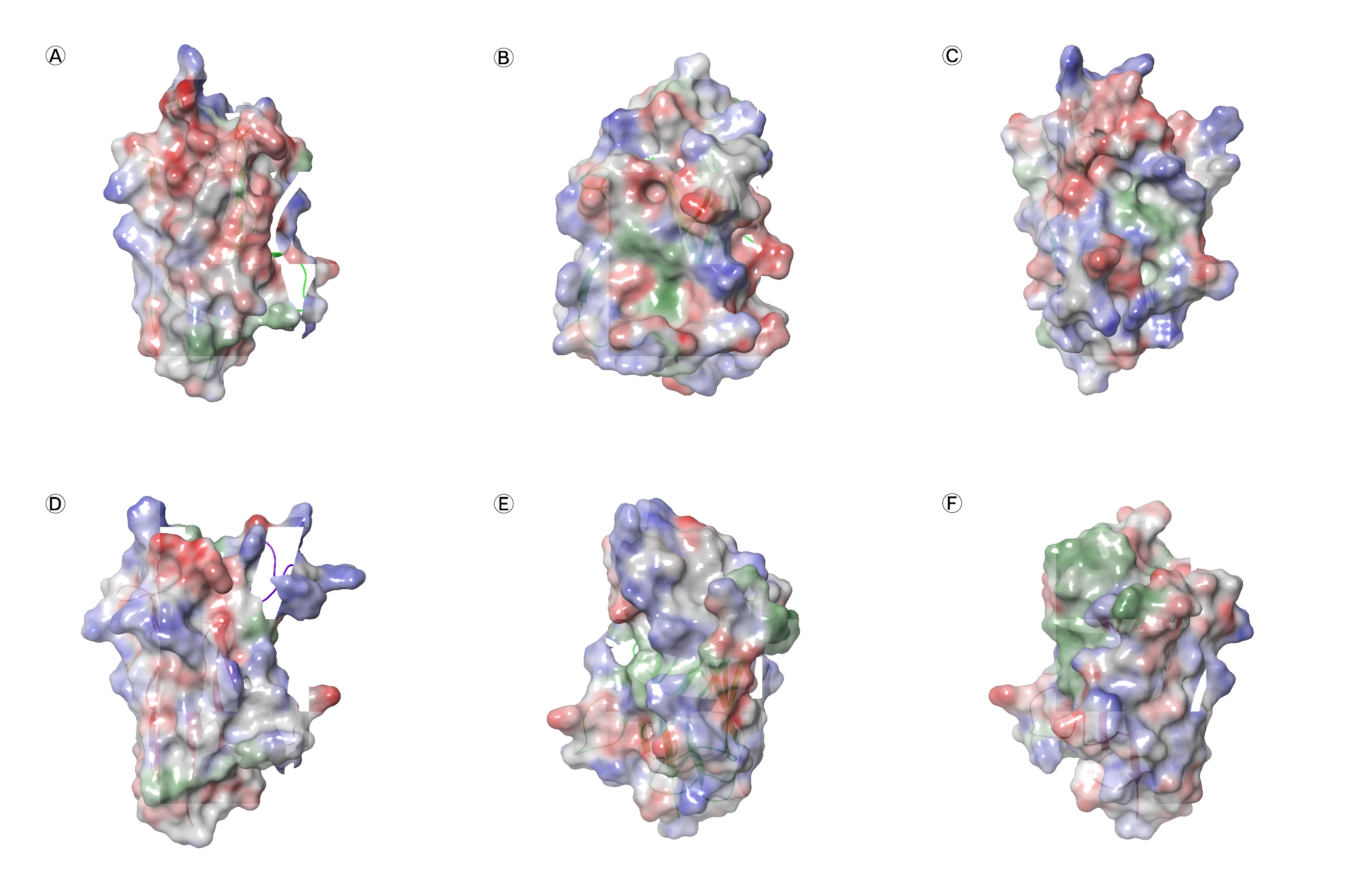


Figure S5. Patch profiles on surfaces of constructed nanobodies. Surfaces indicate the patches of nanobodies A) ECSb1, B) ECSb2, C) ECSb3, D) ECSb4, E) ECSb5, and F) SR6c3 in blue, red, and green colors showing the positively charged, negatively charged and hydrophobic patches, respectively. The intensity of the color indicates the contribution of each residue to the affiliated patch having specific energy (kcal/mol), with darker color indicating higher contributing energy and vice versa.


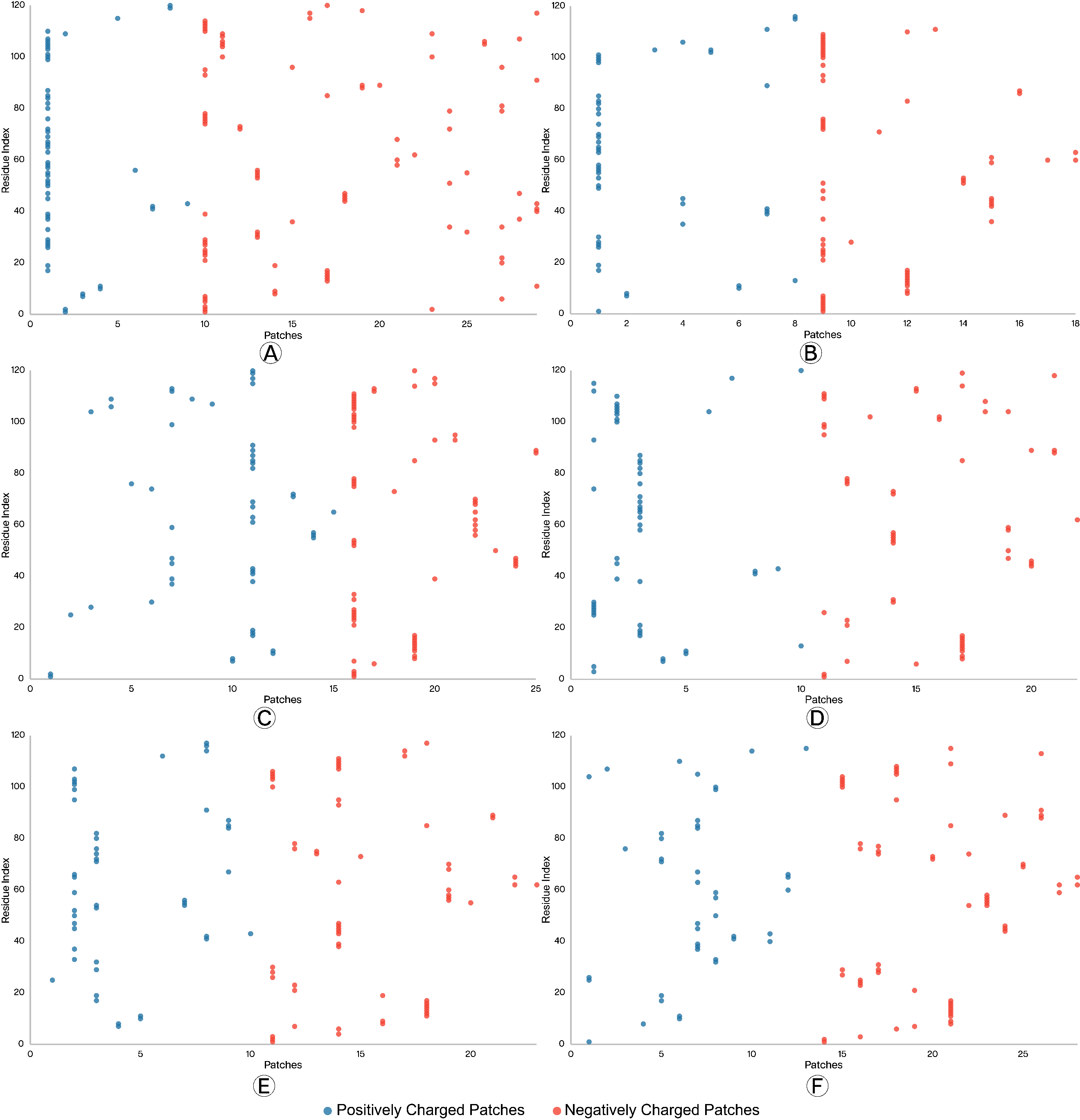


Figure S6. Distribution of residues forming distinct surface patches in Nbs (ECSbs and SR6c3). Residues in (A) ECSb1, (B) ECSb2, (C) ECSb3, (D) ECSb4, (E) ECSb5, and (F) SR6c3 are categorized based on their contribution to positively charged, negatively charged, and hydrophobic surface patches. The horizontal axis indicates the number of patches present in each nanobody, while the vertical axis denotes the specific residues. Blue spheres represent residues within positively charged patches, red spheres indicate residues within negatively charged patches, and green spheres denote residues contributing to hydrophobic patches.


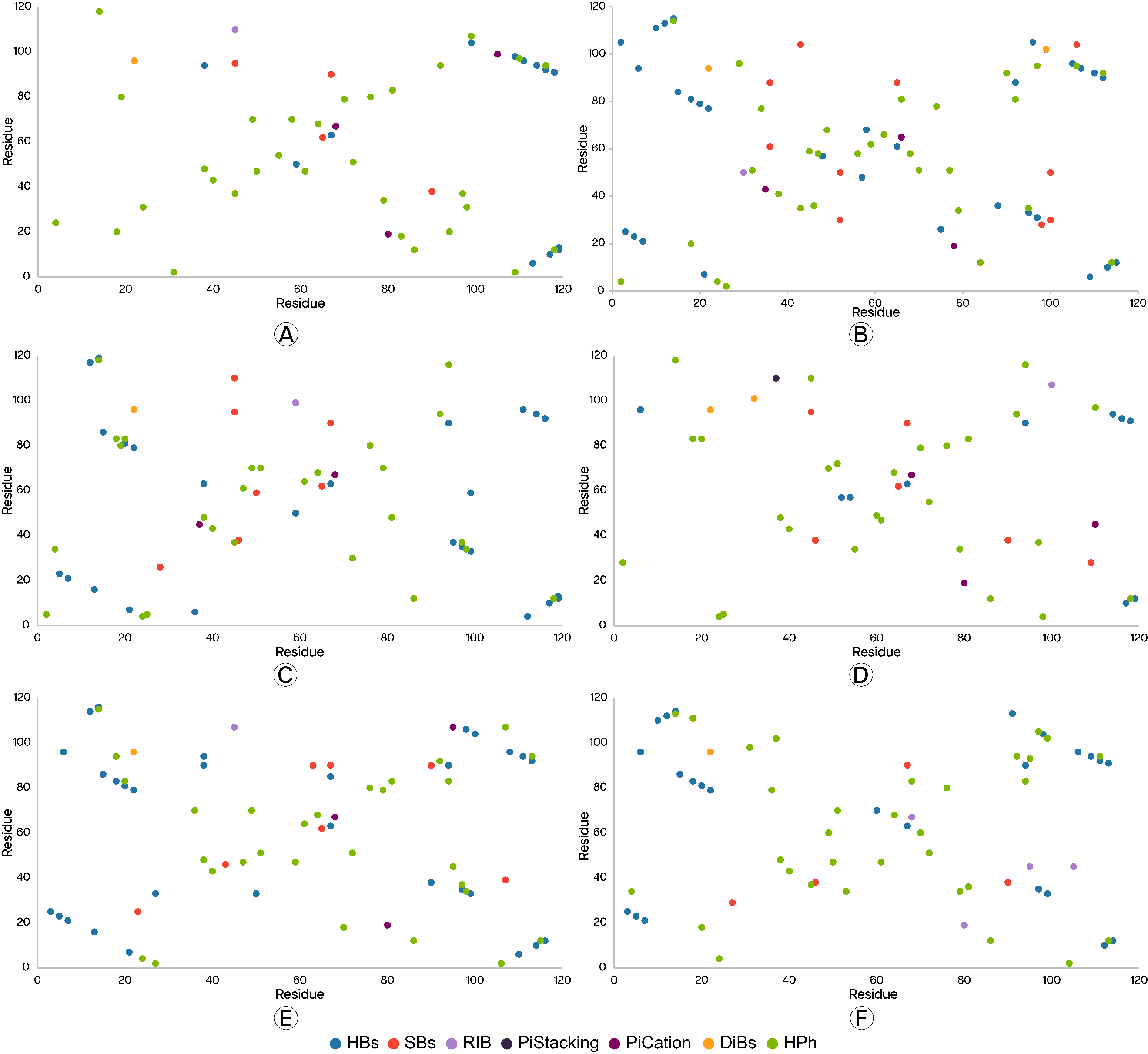


Figure S7. Intramolecular interactions in Nb (ECSbs and SR6c3) structures. A) ECSb1, B) ECSb2, C) ECSb3, D) ECSb4, E) ECSb5, and F) SR6c3 Residues involved in various intramolecular interactions within the Nb structures. Distinct colors represent different types of interactions, including hydrogen bonds (HBs), salt bridges (SBs), repulsive ionic bonding (RIB), disulfide bonds (DiBs), π-π stacking (Pi stacking), π-cation (Pi Cation) interactions, contributing to the overall stability and conformation of the nanobody. The residues are plotted on both axes, indicating pairwise interactions.


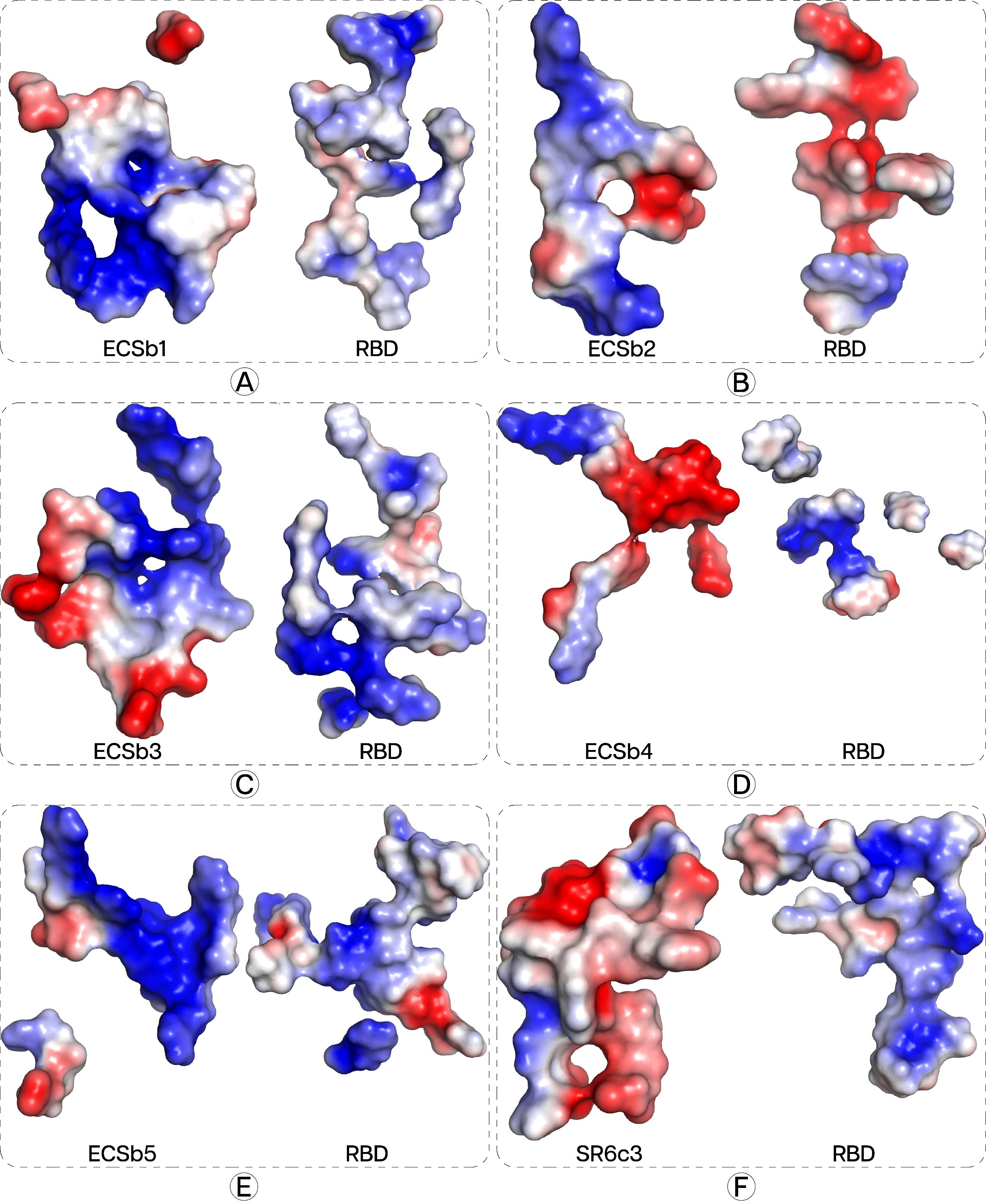


**Figure S8.** Electrostatic potential maps of the binding interfaces in Nb-RBD complexes are presented. The intensity of the coloration indicates the charge density across the electrostatic maps, with blue representing positive charge and red representing negative charge within a range of ±2 kcal/mol·e. Panels A) ECSb1-RBD, B) ECSb2-RBD, C) ECSb3-RBD, D) ECSb4-RBD, E) ECSb5-RBD, and F) SR6c3-RBD illustrate the respective Nb-RBD interactions.

Table S1. Comparison of pre-refinement and post-refinement structural issues in RBD structure. A detailed comparison of structural issues identified in the receptor-binding domain (RBD) before and after structural refinement. The data compares various backbone and sidechain dihedrals, unusual B-factors, bond angle deviations, improper torsions, and steric clashes observed in pre-refinement stage with relatively less issues in post-refinement structure. The G-factor summary indicates several disallowed conformations that persisted post-refinement, despite overall improvements.

| Pre-Refinement Structural Issues | | | | | | Post-Refinement Structural Issues | | | | |
| --- | --- | --- | --- | --- | --- | --- | --- | --- | --- | --- |
| Backbone Dihedrals | **Residue** | **Phi** | **Psi** | **G-Factor** |  | **Backbone Dihedrals** | **Residue** | **Phi** | **Psi** | **G-Factor** |
|  | Pro 337 | -118.64 | 50.66 | Disallowed |  |  | Gly 381 | 67.53 | -0.22 | Disallowed |
|  | Phe 338 | -62.99 | -0.56 | Disallowed |  |  | Gly 482 | 85.03 | -3.53 | Disallowed |
|  | Gly 339 | -41.94 | -85.15 | Disallowed |  |  | Phe 497 | -23.91 | 94.48 | Disallowed |
|  | Cys 361 | -163.85 | -155.38 | Disallowed |  | **Sidechain Dihedrals** | **Residue** | **Chi1** | **Chi2** | **G-Factor** |
|  | Ala 372 | -76.36 | -4.55 | Disallowed |  |  | Leu 387 | 67.57 | 144.07 | Disallowed |
|  | Lys 386 | -107.66 | -1.7 | Disallowed |  |  | Asn 388 | 30.87 | -84.14 | Disallowed |
|  | Leu 387 | 49.82 | -160.05 | Disallowed |  | **G-factor Summary** | **Residue** | **Backbone** | **Sidechain** | **Total** |
|  | Phe 392 | 66.24 | 150.71 | Disallowed |  |  | Gly 381 | Disallowed | Disallowed |  |
|  | Asp 442 | -115.47 | -4.81 | Disallowed |  |  | Leu 387 | -9.772 | Disallowed | Disallowed |
|  | Tyr 505 | -97.08 | -0.81 | Disallowed |  |  | Asn 388 | -8.903 | Disallowed | Disallowed |
|  | Leu 517 | 80.35 | -69.03 | Disallowed |  |  | Gly 482 | Disallowed | Disallowed |  |
|  | Leu 518 | 38.62 | -161.09 | Disallowed |  |  | Phe 497 | Disallowed | -5.097 | Disallowed |
| Sidechain Dihedrals | **Residue** | **Chi1** | **Chi2** | **G-Factor** |  |  |  |  |  |  |
|  | Asn 448 | -170.21 | -3.79 | Disallowed |  |  |  |  |  |  |
|  | Asp 467 | 177.43 | -2.88 | Disallowed |  |  |  |  |  |  |
| Unusual B-factors | **Residue** | **Backbone** | **BBStdDev** | **Sidechain** | **SCStdDev** |  |  |  |  |  |
|  | Thr 385 | 116.83 | 3.2 | 122.23 | 19.61 |  |  |  |  |  |
|  | Leu 518 | 101.11 | 29.27 | 92.77 | 26.97 |  |  |  |  |  |
|  | Hid 519 | 107.75 | 7.45 | 121.91 | 13.8 |  |  |  |  |  |
|  | Ala 520 | 96.7 | 5.34 | 100.21 | 0 |  |  |  |  |  |
| Steric Clashes | **Atom** | **Distance** | **Min** | **Allowed Delta** |  |  |  |  |  |  |
|  | Cys 336: SG-Cys 361: SG | 2.03 | 3.6 | 1.57 |  |  |  |  |  |  |
|  | Asn 343: O-Asn 344: O | 2.7 | 3.22 | 0.52 |  |  |  |  |  |  |
|  | Val 367: O-Val 368: O | 2.72 | 3.22 | 0.5 |  |  |  |  |  |  |
|  | Cys 379: SG-Cys 432: SG | 2.06 | 3.6 | 1.54 |  |  |  |  |  |  |
|  | Cys 480: SG-Cys 488: SG | 2.05 | 3.6 | 1.55 |  |  |  |  |  |  |
|  | Gly 496: O-Gly 497: O | 2.7 | 3.22 | 0.52 |  |  |  |  |  |  |
| Bond Angle Deviations | **Angle** | **Deviation** |  |  |  |  |  |  |  |  |
|  | Leu 387: CA-CB-CG | 15.9 |  |  |  |  |  |  |  |  |
|  | Leu 518: CA-CB-CG | 11.7 |  |  |  |  |  |  |  |  |
| Improper Torsions | **Residue** | **RMS Deviation** |  |  |  |  |  |  |  |  |
|  | Asn 354 | 17.28 |  |  |  |  |  |  |  |  |
|  | Phe 377 | 21.25 |  |  |  |  |  |  |  |  |
|  | Asn 388 | 11.27 |  |  |  |  |  |  |  |  |

Table S2. Characterization of epitope residues and their pKa values. The table presents charged residues within the AS1, AS2, AS3, CA1, and CA2 epitopes for their charges across a pH range of 2–12, along with their corresponding pKa values.

| Ag | Residues | pKa | 2 | 2.5 | 3 | 3.5 | 4 | 4.5 | 5 | 5.5 | 6 | 6.5 | 7 | 7.5 | 8 | 8.5 | 9 | 9.5 | 10 | 10.5 | 11 | 11.5 | 12 |
| --- | --- | --- | --- | --- | --- | --- | --- | --- | --- | --- | --- | --- | --- | --- | --- | --- | --- | --- | --- | --- | --- | --- | --- |
|  |  |  | pH Scale | | | | | | | | | | | | | | | | | | | | |
| AS1 | TYR473 | 10.1 | 0.0 | 0.0 | 0.0 | 0.0 | 0.0 | 0.0 | 0.0 | 0.0 | 0.0 | 0.0 | 0.0 | 0.0 | 0.0 | 0.0 | -0.1 | -0.2 | -0.4 | -0.7 | -0.9 | -1.0 | -1.0 |
|  | GLU484 | 4.8 | 0.0 | 0.0 | 0.0 | 0.0 | -0.1 | -0.3 | -0.6 | -0.8 | -0.9 | -1.0 | -1.0 | -1.0 | -1.0 | -1.0 | -1.0 | -1.0 | -1.0 | -1.0 | -1.0 | -1.0 | -1.0 |
|  | TYR489 | 9.9 | 0.0 | 0.0 | 0.0 | 0.0 | 0.0 | 0.0 | 0.0 | 0.0 | 0.0 | 0.0 | 0.0 | 0.0 | 0.0 | 0.0 | -0.1 | -0.3 | -0.5 | -0.8 | -0.9 | -1.0 | -1.0 |
|  | TYR495 | 10.0 | 0.0 | 0.0 | 0.0 | 0.0 | 0.0 | 0.0 | 0.0 | 0.0 | 0.0 | 0.0 | 0.0 | 0.0 | 0.0 | 0.0 | -0.1 | -0.2 | -0.5 | -0.7 | -0.9 | -1.0 | -1.0 |
| AS2 | CYX432 | 8.8 | 0.0 | 0.0 | 0.0 | 0.0 | 0.0 | 0.0 | 0.0 | 0.0 | 0.0 | 0.0 | 0.0 | 0.0 | 0.0 | 0.0 | 0.0 | 0.0 | 0.0 | 0.0 | 0.0 | 0.0 | 0.0 |
|  | LYS444 | 10.3 | 1.0 | 1.0 | 1.0 | 1.0 | 1.0 | 1.0 | 1.0 | 1.0 | 1.0 | 1.0 | 1.0 | 1.0 | 1.0 | 1.0 | 1.0 | 0.9 | 0.7 | 0.4 | 0.2 | 0.1 | 0.0 |
| AS3 | TYR369 | 10.6 | 0.0 | 0.0 | 0.0 | 0.0 | 0.0 | 0.0 | 0.0 | 0.0 | 0.0 | 0.0 | 0.0 | 0.0 | 0.0 | 0.0 | 0.0 | -0.1 | -0.2 | -0.4 | -0.7 | -0.9 | -1.0 |
|  | LYS378 | 10.3 | 1.0 | 1.0 | 1.0 | 1.0 | 1.0 | 1.0 | 1.0 | 1.0 | 1.0 | 1.0 | 1.0 | 1.0 | 1.0 | 1.0 | 1.0 | 0.9 | 0.7 | 0.4 | 0.2 | 0.1 | 0.0 |
|  | CYX379 | 8.8 | 0.0 | 0.0 | 0.0 | 0.0 | 0.0 | 0.0 | 0.0 | 0.0 | 0.0 | 0.0 | 0.0 | 0.0 | 0.0 | 0.0 | 0.0 | 0.0 | 0.0 | 0.0 | 0.0 | 0.0 | 0.0 |
|  | TYR380 | 10.2 | 0.0 | 0.0 | 0.0 | 0.0 | 0.0 | 0.0 | 0.0 | 0.0 | 0.0 | 0.0 | 0.0 | 0.0 | 0.0 | 0.0 | -0.1 | -0.2 | -0.4 | -0.7 | -0.9 | -1.0 | -1.0 |
|  | LYS386 | 10.4 | 1.0 | 1.0 | 1.0 | 1.0 | 1.0 | 1.0 | 1.0 | 1.0 | 1.0 | 1.0 | 1.0 | 1.0 | 1.0 | 1.0 | 1.0 | 0.9 | 0.7 | 0.5 | 0.2 | 0.1 | 0.0 |
|  | CYS391 | 9.1 | 0.0 | 0.0 | 0.0 | 0.0 | 0.0 | 0.0 | 0.0 | 0.0 | 0.0 | 0.0 | 0.0 | 0.0 | -0.1 | -0.2 | -0.4 | -0.7 | -0.9 | -1.0 | -1.0 | -1.0 | -1.0 |
| CA1 | TYR449 | 10.1 | 0.0 | 0.0 | 0.0 | 0.0 | 0.0 | 0.0 | 0.0 | 0.0 | 0.0 | 0.0 | 0.0 | 0.0 | 0.0 | 0.0 | -0.1 | -0.2 | -0.4 | -0.7 | -0.9 | -1.0 | -1.0 |
|  | TYR451 | 10.0 | 0.0 | 0.0 | 0.0 | 0.0 | 0.0 | 0.0 | 0.0 | 0.0 | 0.0 | 0.0 | 0.0 | 0.0 | 0.0 | 0.0 | -0.1 | -0.2 | -0.5 | -0.7 | -0.9 | -1.0 | -1.0 |
|  | TYR453 | 10.1 | 0.0 | 0.0 | 0.0 | 0.0 | 0.0 | 0.0 | 0.0 | 0.0 | 0.0 | 0.0 | 0.0 | 0.0 | 0.0 | 0.0 | -0.1 | -0.2 | -0.4 | -0.7 | -0.9 | -1.0 | -1.0 |
|  | ARG454 | 12.3 | 1.0 | 1.0 | 1.0 | 1.0 | 1.0 | 1.0 | 1.0 | 1.0 | 1.0 | 1.0 | 1.0 | 1.0 | 1.0 | 1.0 | 1.0 | 1.0 | 1.0 | 1.0 | 0.9 | 0.8 | 0.6 |
|  | ARG457 | 12.8 | 1.0 | 1.0 | 1.0 | 1.0 | 1.0 | 1.0 | 1.0 | 1.0 | 1.0 | 1.0 | 1.0 | 1.0 | 1.0 | 1.0 | 1.0 | 1.0 | 1.0 | 1.0 | 1.0 | 1.0 | 0.9 |
|  | LYS458 | 10.5 | 1.0 | 1.0 | 1.0 | 1.0 | 1.0 | 1.0 | 1.0 | 1.0 | 1.0 | 1.0 | 1.0 | 1.0 | 1.0 | 1.0 | 1.0 | 0.9 | 0.7 | 0.5 | 0.2 | 0.1 | 0.0 |
|  | LYS462 | 10.4 | 1.0 | 1.0 | 1.0 | 1.0 | 1.0 | 1.0 | 1.0 | 1.0 | 1.0 | 1.0 | 1.0 | 1.0 | 1.0 | 1.0 | 1.0 | 0.9 | 0.7 | 0.4 | 0.2 | 0.1 | 0.0 |
|  | GLU465 | 4.0 | 0.0 | 0.0 | -0.1 | -0.3 | -0.5 | -0.8 | -0.9 | -1.0 | -1.0 | -1.0 | -1.0 | -1.0 | -1.0 | -1.0 | -1.0 | -1.0 | -1.0 | -1.0 | -1.0 | -1.0 | -1.0 |
|  | ARG466 | 12.5 | 1.0 | 1.0 | 1.0 | 1.0 | 1.0 | 1.0 | 1.0 | 1.0 | 1.0 | 1.0 | 1.0 | 1.0 | 1.0 | 1.0 | 1.0 | 1.0 | 1.0 | 1.0 | 1.0 | 0.9 | 0.7 |
| CA2 | CYX488 | 8.8 | 0.0 | 0.0 | 0.0 | 0.0 | 0.0 | 0.0 | 0.0 | 0.0 | 0.0 | 0.0 | 0.0 | 0.0 | 0.0 | 0.0 | 0.0 | 0.0 | 0.0 | 0.0 | 0.0 | 0.0 | 0.0 |
|  | TYR495 | 10.1 | 0.0 | 0.0 | 0.0 | 0.0 | 0.0 | 0.0 | 0.0 | 0.0 | 0.0 | 0.0 | 0.0 | 0.0 | 0.0 | 0.0 | -0.1 | -0.2 | -0.5 | -0.7 | -0.9 | -1.0 | -1.0 |

Table S3. Engineered framework regions (FRs) and complementarity-determining regions (CDRs) in ECSbs sequences with template sequence of SR6c3.The complete engineered sequences of framework regions (FRs) and complementarity-determining regions (CDRs) for ECSbs sequences, along with the template sequence of SR6c3.

| Name | FR1 | CDR1 | FR2 | CDR2 | FR3 | CDR3 | FR4 |
| --- | --- | --- | --- | --- | --- | --- | --- |
| SR6c3 | EVQLVESGGGLVQAGDSLRLSCAASG | DCHVLWR | MGWFRQAPGKEREFVAAIS | LDGQF | TNYADSVRGRFSISADSAKNTVYLQMNSLKPEDTAVYYCAA | LPFL | DYWGQGTQVTVSS |
| ECSb1 | EVQLVESGGGLVQAGDSLRLSCDADR | ERDPYPQ | MGSFRQAPGKEREFVARIS | DRYNR | RNDADSVRGRFSISADSAKNTVYLQMNSLKPEDTADYECAA | RPSCNQYPM | DYRGDGTQVTVSS |
| ECSb2 | EVQLVESGGGLVQAGDSLRLSCEADK | QDRRC | MGWFRQAPGKEREFVAEIR | YDQPR | KNYADDVRGRFSISADSAKNTVYLQMNSLKPEDRAEYSCAA | WRCDRCQ | DDRGEGTQVTVSS |
| ECSb3 | EVQLVESGGGLVQAGDSLRLSCDARD | PRCWQSP | MGQFRQAPGKEREFVADIS | SDRPQ | TRDADRVRGRFSISADSAKNTVYLQMNSLKPEDRAEYDCAA | RSDCDRCRS | DYDGQRTQVTVSS |
| ECSb4 | EVQLVESGGGLVQAGDSLRLSCDARE | QRPRSCP | MGQFRQAPGKEREFVADIS | DNWDD | TERADSVRGRFSISADSAKNTVYLQMNSLKPEDTARYDCAA | PRCDRWRDR | DEWGRGTQVTVSS |
| ECSb5 | EVQLVESGGGLVQAGDSLRLSCEARD | RDPSCRQ | MGWFRDAPGKEREFVARIS | QPQWT | DREADDVRGRFSISADSAKNTVYLQMNSLKPEDRADYYCAA | RERCRQ | DYRGEGTQVTVSS |

Table S4. Dihedral angles and G-factor summary of Nbs (ECSbs and SR6c3) post-structural refinement. The backbone/sidechain dihedral angles and corresponding G-factor summaries for ECSbs and SR6c3. Despite structural refinement, several structural issues remain, particularly with sidechain accessibility and backbone conformation, as indicated by the disallowed G-factors. These unresolved structural irregularities may affect the stability and functionality of the Nbs.

| Nbs | Residue |  | Chi1/Phi | Chi2/Psi | G-Factor | G-factor Summary | Backbone | Sidechain | Total |
| --- | --- | --- | --- | --- | --- | --- | --- | --- | --- |
| ECSb1 | **Asn 59** | Sidechain Dihedrals | 158.24 | -134.81 | Disallowed | **Asn 59** | -6.418 | Disallowed | Disallowed |
|  | **Asp 60** | Sidechain Dihedrals | -42.84 | 86.81 | Disallowed | **Asp 60** | -6.182 | Disallowed | Disallowed |
| ECSb2 | **Val 62** | Backbone Dihedrals | -102.83 | -2.46 | Disallowed | **Val 62** | Disallowed | -2.159 | Disallowed |
|  | **Asp 88** | Backbone Dihedrals | -80.77 | -1.04 | Disallowed | **Asp 88** | Disallowed | -6.381 | Disallowed |
| ECSb3 | **Tyr 109** | Backbone Dihedrals | -66.3 | 80.76 | Disallowed | **Tyr 109** | Disallowed | -4.141 | Disallowed |
| ECSb4 | **Val 64** | Backbone Dihedrals | -121.56 | -2.8 | Disallowed | **Pro 29** | -8.445 | Disallowed | Disallowed |
|  | **Pro 29** | Sidechain Dihedrals | -61.03 | 10 | Disallowed | **Val 64** | Disallowed | -2.159 | Disallowed |
|  | **Arg 107** | Sidechain Dihedrals | -126.22 | 60 | Disallowed | **Arg 107** | -7.969 | Disallowed | Disallowed |
| ECSb5 | **Gly 8** | Backbone Dihedrals | 95.78 | -1.96 | Disallowed | **Gly 8** | Disallowed | Disallowed |  |
|  | **Trp 56** | Backbone Dihedrals | -62.73 | 57.65 | Disallowed | **Trp 56** | Disallowed | -5.464 | Disallowed |
|  | **Asp 90** | Backbone Dihedrals | -80.42 | -1.08 | Disallowed | **Asp 90** | Disallowed | -6.381 | Disallowed |
| SR6c3 | **Gly 8** | Backbone Dihedrals | 95.78 | -1.96 | Disallowed | **Gly 8** | Disallowed | Disallowed |  |
|  | **Trp 56** | Backbone Dihedrals | -62.73 | 57.65 | Disallowed | **Trp 56** | Disallowed | -5.464 | Disallowed |
|  | **Asp 90** | Backbone Dihedrals | -80.42 | -1.08 | Disallowed | **Asp 90** | Disallowed | -6.381 | Disallowed |

Table S5. Hotspot residues for post-translational modifications in Nbs (ECSbs and SR6c3). The hotspot residues in ECSbs and SR6c3 and their sidechain accessibility (SCA) percentage for various post-translational modifications, including deamidation (Deam.), oxidation (Oxid.), glycation (Glyc.), proteolysis (Prot.), Asp isomerization (AIS), and free cysteine sites (FCS). The data provides insights into regions vulnerable to specific chemical modifications across different nanobody structures.

| Residue | SCA | Deam. | Oxid. | Glyc. | Prot. | AIS | FCS | Residue | SCA | Deam. | Oxid. | Glyc. | Prot. | AIS | FCS |
| --- | --- | --- | --- | --- | --- | --- | --- | --- | --- | --- | --- | --- | --- | --- | --- |
| ECSb1 | | | | | | | | **ECSb2** | | | | | | | |
| Tyr105 | 51.80% |  | ✔ |  |  |  |  | **Gln27** | 53.40% | ✔ |  |  |  |  |  |
| Asn59 | 43.70% | ✔ |  |  |  |  |  | **Tyr58** | 20.00% |  | ✔ |  |  |  |  |
| Gln104 | 38.80% | ✔ |  |  |  |  |  | **Asn82** | 37.50% | ✔ |  |  |  |  |  |
| Tyr55 | 40.60% |  | ✔ |  |  |  |  | **Tyr78** | 24.00% |  | ✔ |  |  |  |  |
| Asn84 | 38.60% | ✔ |  |  |  |  |  | **Asn57** | 36.40% | ✔ |  |  |  |  |  |
| Tyr80 | 25.20% |  | ✔ |  |  |  |  | **Gln80** | 32.20% | ✔ |  |  |  |  |  |
| Gln82 | 31.70% | ✔ |  |  |  |  |  | **Asp100** | 51.50% |  |  |  | ✔ |  |  |
| Gln39 | 40.20% | ✔ |  |  |  |  |  | **Asp28** | 53.50% |  |  |  | ✔ |  |  |
| Tyr109 | 27.70% |  | ✔ |  |  |  |  | **Gln103** | 62.70% | ✔ |  |  |  |  |  |
| Asp112 | 65.10% |  |  |  | ✔ | ✔ |  | **Gln103** | 62.70% | ✔ |  |  |  |  |  |
| Asp25 | 57.00% |  |  |  | ✔ |  |  | **Gln13** | 43.40% | ✔ |  |  |  |  |  |
| Asp23 | 45.60% |  |  |  | ✔ |  |  | **Asp105** | 39.20% |  |  |  | ✔ |  |  |
| Asp93 | 40.70% |  |  |  | ✔ |  |  | **Asp100** | 51.50% |  |  |  | ✔ |  |  |
| Gln39 | 40.20% | ✔ |  |  |  |  |  | **Asp25** | 33.40% |  |  |  | ✔ |  |  |
| Asp108 | 39.20% |  |  |  | ✔ |  |  | **Gln103** | 62.70% | ✔ |  |  |  |  |  |
| Tyr105 | 51.80% |  | ✔ |  |  |  |  | **Asp104** | 25.90% |  |  |  | ✔ | ✔ |  |
| Gln104 | 38.80% | ✔ |  |  |  |  |  | **Gln37** | 42.30% | ✔ |  |  |  |  |  |
| Tyr109 | 27.70% |  | ✔ |  |  |  |  | **Gln27** | 53.40% | ✔ |  |  |  |  |  |
| Asp73 | 33.80% |  |  |  | ✔ | ✔ |  | **Trp97** | 21.60% |  | ✔ |  |  |  |  |
| Asp53 | 55.00% |  |  |  | ✔ |  |  | **Tyr51** | 33.20% |  | ✔ |  |  |  |  |
| Tyr55 | 40.60% |  | ✔ |  |  |  |  | **Asp28** | 53.50% |  |  |  | ✔ |  |  |
| Asp16 | 42.70% |  |  |  | ✔ | ✔ |  | **Asp71** | 34.70% |  |  |  | ✔ | ✔ |  |
| Gln13 | 45.30% | ✔ |  |  |  |  |  | **Asp16** | 41.90% |  |  |  | ✔ | ✔ |  |
| Asp60 | 25.50% |  |  |  | ✔ |  |  | **Gln13** | 43.40% | ✔ |  |  |  |  |  |
| Asp62 | 56.10% |  |  |  | ✔ | ✔ |  | **Asp52** | 31.40% |  |  |  | ✔ |  |  |
| Tyr109 | 27.70% |  | ✔ |  |  |  |  | **Tyr51** | 33.20% |  | ✔ |  |  |  |  |
| Tyr55 | 40.60% |  | ✔ |  |  |  |  | **Asp61** | 48.60% |  |  |  | ✔ |  |  |
| Tyr105 | 51.80% |  | ✔ |  |  |  |  | **Asp60** | 57.70% |  |  |  | ✔ | ✔ |  |
| ECSb3 | | | | | | | | **Asp60** | 57.70% |  |  |  | ✔ | ✔ |  |
| Tyr109 | 29.20% |  | ✔ |  |  |  |  | **Tyr51** | 33.20% |  | ✔ |  |  |  |  |
| Gln39 | 36.60% | ✔ |  |  |  |  |  | **Tyr58** | 20.00% |  | ✔ |  |  |  |  |
| Tyr109 | 29.20% |  | ✔ |  |  |  |  | **Trp97** | 21.60% |  | ✔ |  |  |  |  |
| Asn84 | 38.60% | ✔ |  |  |  |  |  | **ECSb4** | | | | | | | |
| Gln82 | 29.00% | ✔ |  |  |  |  |  | **Trp104** | 49.90% |  | ✔ |  |  |  |  |
| Asp108 | 70.30% |  |  |  | ✔ |  |  | **Trp110** | 31.00% |  | ✔ |  |  |  |  |
| Asp103 | 65.70% |  |  |  | ✔ |  |  | **Gln39** | 35.70% | ✔ |  |  |  |  |  |
| Asp110 | 42.10% |  |  |  | ✔ | ✔ |  | **Tyr80** | 25.80% |  | ✔ |  |  |  |  |
| Asp54 | 52.10% |  |  |  | ✔ |  |  | **Asn84** | 38.10% | ✔ |  |  |  |  |  |
| Asp101 | 50.00% |  |  |  | ✔ |  |  | **Gln82** | 32.40% | ✔ |  |  |  |  |  |
| Asp23 | 36.10% |  |  |  | ✔ |  |  | **Trp104** | 49.90% |  | ✔ |  |  |  |  |
| Asp26 | 23.40% |  |  |  | ✔ |  |  | **Gln13** | 44.20% | ✔ |  |  |  |  |  |
| Gln31 | 39.70% | ✔ |  |  |  |  |  | **Trp110** | 31.00% |  | ✔ |  |  |  |  |
| Cys102 | 24.00% |  | ✔ |  |  |  | ✔ | **Asp23** | 32.40% |  |  |  | ✔ |  |  |
| Tyr109 | 29.20% |  | ✔ |  |  |  |  | **Asp102** | 52.00% |  |  |  | ✔ |  |  |
| Asp73 | 32.60% |  |  |  | ✔ | ✔ |  | **Asp56** | 50.60% |  |  |  | ✔ | ✔ |  |
| Asp16 | 40.90% |  |  |  | ✔ | ✔ |  | **Asp53** | 48.50% |  |  |  | ✔ |  |  |
| Gln13 | 45.30% | ✔ |  |  |  |  |  | **Asp57** | 48.80% |  |  |  | ✔ | ✔ |  |
| Gln39 | 36.60% | ✔ |  |  |  |  |  | **Asp73** | 34.20% |  |  |  | ✔ | ✔ |  |
| Asp60 | 35.50% |  |  |  | ✔ |  |  | **Asp102** | 52.00% |  |  |  | ✔ |  |  |
| Asp62 | 52.20% |  |  |  | ✔ |  |  | **Asp16** | 41.50% |  |  |  | ✔ | ✔ |  |
| ECSb5 | | | | | | | | **Gln13** | 44.20% | ✔ |  |  |  |  |  |
| Gln33 | 26.10% | ✔ |  |  |  |  |  | **Asp108** | 29.50% |  |  |  | ✔ |  |  |
| Cys102 | 45.90% |  | ✔ |  |  |  | ✔ | **Trp104** | 49.90% |  | ✔ |  |  |  |  |
| Tyr80 | 23.70% |  | ✔ |  |  |  |  | **Asp50** | 28.90% |  |  |  | ✔ |  |  |
| Gln82 | 31.90% | ✔ |  |  |  |  |  | **Trp104** | 49.90% |  | ✔ |  |  |  |  |
| Trp56 | 58.40% |  | ✔ |  |  |  |  | **Asp62** | 56.70% |  |  |  | ✔ | ✔ |  |
| Asn84 | 38.10% | ✔ |  |  |  |  |  | **Trp104** | 49.90% |  | ✔ |  |  |  |  |
| Asp26 | 53.20% |  |  |  | ✔ |  |  | **Trp110** | 31.00% |  | ✔ |  |  |  |  |
| Asp105 | 55.20% |  |  |  | ✔ |  |  | **SR6c3** | | | | | | | |
| Asp28 | 37.60% |  |  |  | ✔ |  |  | **Tyr104** | 32.70% |  | ✔ |  |  |  |  |
| Gln104 | 48.90% | ✔ |  |  |  |  |  | **Gln107** | 60.30% | ✔ |  |  |  |  |  |
| Tyr106 | 27.70% |  | ✔ |  |  |  |  | **Tyr80** | 22.00% |  | ✔ |  |  |  |  |
| Asp93 | 38.60% |  |  |  | ✔ |  |  | **Gln82** | 32.50% | ✔ |  |  |  |  |  |
| Asp39 | 36.00% |  |  |  | ✔ |  |  | **Asn84** | 36.60% | ✔ |  |  |  |  |  |
| Asp63 | 51.20% |  |  |  | ✔ |  |  | **Gln39** | 28.60% | ✔ |  |  |  |  |  |
| Asp73 | 34.30% |  |  |  | ✔ | ✔ |  | **Trp105** | 27.10% |  | ✔ |  |  |  |  |
| Asp16 | 41.30% |  |  |  | ✔ | ✔ |  | **Asn59** | 51.00% | ✔ |  |  |  |  |  |
| Gln13 | 44.20% | ✔ |  |  |  |  |  | **Trp32** | 49.60% |  | ✔ |  |  |  |  |
| Asp58 | 41.60% |  |  |  | ✔ |  |  | **Tyr60** | 22.60% |  | ✔ |  |  |  |  |
| Trp56 | 58.40% |  | ✔ |  |  |  |  | **Asp103** | 49.90% |  |  |  | ✔ |  |  |
| Asp62 | 55.00% |  |  |  | ✔ | ✔ |  | **Asp27** | 56.10% |  |  |  | ✔ |  |  |
| Asp62 | 55.00% |  |  |  | ✔ | ✔ |  | **His29** | 60.90% |  | ✔ |  |  |  |  |
| Trp56 | 58.40% |  | ✔ |  |  |  |  | **Tyr104** | 32.70% |  | ✔ |  |  |  |  |
|  |  |  |  |  |  |  |  | **Cys28** | 34.20% |  | ✔ |  |  |  | ✔ |
|  |  |  |  |  |  |  |  | **His29** | 60.90% |  | ✔ |  |  |  |  |
|  |  |  |  |  |  |  |  | **Gln107** | 60.30% | ✔ |  |  |  |  |  |
|  |  |  |  |  |  |  |  | **Trp105** | 27.10% |  | ✔ |  |  |  |  |
|  |  |  |  |  |  |  |  | **Asp73** | 33.50% |  |  |  | ✔ | ✔ |  |
|  |  |  |  |  |  |  |  | **Asp16** | 41.10% |  |  |  | ✔ | ✔ |  |
|  |  |  |  |  |  |  |  | **Gln13** | 45.00% | ✔ |  |  |  |  |  |
|  |  |  |  |  |  |  |  | **Asp54** | 49.50% |  |  |  | ✔ | ✔ |  |
|  |  |  |  |  |  |  |  | **Asp54** | 49.50% |  |  |  | ✔ | ✔ |  |
|  |  |  |  |  |  |  |  | **Asp62** | 55.50% |  |  |  | ✔ | ✔ |  |
|  |  |  |  |  |  |  |  | **Asn59** | 51.00% | ✔ |  |  |  |  |  |
|  |  |  |  |  |  |  |  | **Asp62** | 55.50% |  |  |  | ✔ | ✔ |  |
|  |  |  |  |  |  |  |  | **Trp32** | 49.60% |  | ✔ |  |  |  |  |
|  |  |  |  |  |  |  |  | **Trp105** | 27.10% |  | ✔ |  |  |  |  |
|  |  |  |  |  |  |  |  | **Tyr104** | 32.70% |  | ✔ |  |  |  |  |
|  |  |  |  |  |  |  |  | **Cys28** | 34.20% |  | ✔ |  |  |  | ✔ |

**Table S6.** Different interaction types including hydrogen bonds (H-Bond), salt bridges, π-anion, π-π stacking, π-cation interactions, van der Waals (vdW) interactions, and repulsive ionic bonds and their network diversity for each Nb-RBD complex.

| Complex | H-Bond | Salt Bridges | π-Anion | π-π Stacking | π-Cation | vdW | Repulsive Ionic | Total |
| --- | --- | --- | --- | --- | --- | --- | --- | --- |
| ECSb1-RBD | 6 (Narrow) | 1 | 0 | 0 | 0 | 5 (Broad) | 0 | 11 |
| ECSb2-RBD | 10 (Narrow) | 4 (Narrow) | 3 (Narrow) | 4 (Narrow) | 0 | 27 (Broad) | 1 | 44 |
| ECSb3-RBD | 17 (Narrow) | 2 (Narrow) | 0 | 5 (Narrow) | 1 | 13 (Broad) | 0 | 38 |
| ECSb4-RBD | 14 (Narrow) | 1 | 0 | 0 | 5 (Narrow) | 39 (Broad) | 1 | 59 |
| ECSb5-RBD | 13 (Narrow) | 1 | 0 | 2 (Narrow) | 2 (Narrow) | 5 (Broad) | 0 | 23 |
| SR6c3-RBD | 14 (Narrow) | 0 | 0 | 5 (Narrow) | 0 | 28 (Broad) | 0 | 47 |

Table S7. Binding energy components including electrostatic (ΔGCoulomb), covalent (ΔGCovalent), hydrogen bonding (ΔGHbond), lipophilic (ΔGLipo), solvation (ΔGSolvGB), and van der Waals (ΔGvdW) interactions calculated at intervals of 20 ns for each Nb-RBD complex, along with their average across 100 ns of simulation time.

| Time (ns) | ΔG | ΔG_Coulomb_ | ΔG_Covalent_ | ΔG_Hbond_ | ΔG_Lipo_ | ΔG_SolvGB_ | Time (ns) | ΔG | ΔG_Coulomb_ | ΔG_Covalent_ | ΔG_Hbond_ | ΔG_Lipo_ | ΔG_SolvGB_ |
| --- | --- | --- | --- | --- | --- | --- | --- | --- | --- | --- | --- | --- | --- |
| ECSb1-RBD | | | | | | | **ECSb4-RBD** | | | | | | |
| 0 | 29.75 | 5.46 | -1.03 | -18.33 | -26.80 | -47.50 | **0** | 102.39 | -13.22 | -2.51 | -81.03 | -61.29 | -138.66 |
| 20 | 40.29 | 20.85 | -0.24 | -18.40 | -1.62 | -67.17 | **20** | 225.25 | 57.47 | -6.01 | -132.14 | -93.86 | -276.58 |
| 40 | 28.94 | -6.56 | 0.01 | -17.14 | -17.35 | -41.24 | **40** | -4.20 | -39.32 | 0.16 | 3.06 | 0.11 | -121.97 |
| 60 | -5.81 | 1.54 | -8.35 | -28.11 | 38.33 | -65.89 | **60** | -6.55 | -120.76 | 0.34 | 5.51 | -1.58 | -56.19 |
| 80 | -15.54 | 28.98 | -4.87 | -38.75 | 38.07 | -79.44 | **80** | -11.01 | -114.03 | -0.43 | 10.54 | -3.07 | -47.13 |
| 100 | -2.44 | -17.77 | -1.42 | -1.75 | 31.74 | -36.26 | **100** | -11.72 | -106.33 | 2.91 | 20.97 | -15.19 | -59.38 |
| Avg | 12.53 | 5.42 | -2.65 | -20.41 | 10.40 | -56.25 | **Avg** | 49.02 | -56.03 | -0.92 | -28.85 | -29.15 | -116.65 |
| ECSb2-RBD | | | | | | | **ECSb5-RBD** | | | | | | |
| 0 | 14.64 | 16.10 | -15.11 | -82.99 | 28.84 | -208.91 | **0** | 25.00 | 10.12 | -9.30 | -58.60 | 27.30 | -107.64 |
| 20 | 40.57 | 158.71 | -14.26 | -106.87 | -0.47 | -207.89 | **20** | 40.07 | 39.84 | -7.09 | -46.66 | -6.71 | -106.36 |
| 40 | 56.72 | 44.56 | -14.54 | -102.17 | 5.70 | -205.14 | **40** | -19.32 | 118.13 | -7.43 | -35.43 | 56.59 | -61.52 |
| 60 | 103.81 | 207.60 | -5.13 | -85.11 | -26.71 | 331.74 | **60** | 9.94 | 25.68 | -12.71 | -49.88 | 18.84 | -79.05 |
| 80 | 79.73 | 52.22 | -20.11 | -96.21 | 26.26 | -202.02 | **80** | 29.07 | 30.33 | -8.00 | -66.28 | 25.61 | -126.74 |
| 100 | 75.94 | 63.39 | -7.41 | -114.12 | 27.80 | -197.54 | **100** | 14.00 | 50.06 | -7.84 | -45.02 | 24.37 | -82.37 |
| Avg | 61.90 | 90.43 | -12.76 | -97.91 | 10.24 | -114.96 | **Avg** | 16.46 | 45.69 | -8.73 | -50.31 | 24.33 | -93.95 |
| ECSb3-RBD | | | | | | | **SR6c3-RBD** | | | | | | |
| 0 | -61.92 | 22.12 | -20.41 | -64.02 | 78.67 | -190.94 | **0** | 78.25 | 27.37 | -20.01 | -77.41 | -76.00 | -153.21 |
| 20 | 15.31 | -10.11 | -16.69 | -38.55 | 52.76 | -161.92 | **20** | 181.02 | 7.09 | -1.85 | -62.63 | -102.17 | -148.18 |
| 40 | 7.86 | -31.78 | -16.58 | -32.15 | 72.41 | -100.14 | **40** | 165.43 | 24.17 | -16.92 | -23.18 | -87.37 | -96.94 |
| 60 | 8.51 | 35.35 | -13.85 | -54.66 | 37.02 | -128.31 | **60** | 208.80 | 27.97 | -4.25 | -63.95 | -120.52 | -143.95 |
| 80 | 49.62 | -3.03 | -11.85 | -23.12 | 22.64 | -87.08 | **80** | 166.84 | 14.94 | -7.22 | -58.34 | -89.57 | -150.85 |
| 100 | 83.07 | 130.09 | -4.83 | -73.51 | 23.67 | -109.98 | **100** | 253.33 | 240.69 | 4.19 | -54.00 | -102.58 | 209.66 |
| Avg | 17.08 | 23.77 | -14.04 | -47.67 | 47.86 | -129.73 | **Avg** | 175.61 | 57.04 | -7.67 | -56.58 | -96.37 | -80.58 |
